# Human papillomaviruses sensitize cells to DNA damage induced apoptosis by targeting the innate immune sensor cGAS

**DOI:** 10.1371/journal.ppat.1010725

**Published:** 2022-07-25

**Authors:** Elona Gusho, Laimonis A. Laimins

**Affiliations:** Department of Microbiology-Immunology, Northwestern University Feinberg School of Medicine, Chicago, Illinois, United States of America; University of North Carolina at Chapel Hill, UNITED STATES

## Abstract

The cyclic GMP-AMP synthase (cGAS) is a critical regulator of the innate immune response acting as a sensor of double-strand DNAs from pathogens or damaged host DNA. Upon activation, cGAS signals through the STING/TBK1/IRF3 pathway to induce interferon expression. Double stranded DNA viruses target the cGAS pathway to facilitate infection. In HPV positive cells that stably maintain viral episomes, the levels of cGAS were found to be significantly increased over those seen in normal human keratinocytes. Furthermore the downstream effectors of the cGAS pathway, STING and IRF3, were fully active in response to signaling from the secondary messenger cGAMP or poly (dA:dT). In HPV positive cells cGAS was detected in both cytoplasmic puncta as well as in DNA damage induced micronuclei. E6 was responsible for increased levels of cGAS that was dependent on inhibition of p53. CRISPR-Cas9 mediated knockout of cGAS prevented activation of STING and IRF3 but had a minimal effect on viral replication. A primary function of cGAS in HPV positive cells was in response to treatment with etoposide or cisplatin which lead to increased levels of H2AX phosphorylation and activation of caspase 3/7 cleavage while having only a minimal effect on activation of homologous recombination repair factors ATM, ATR or CHK2. In HPV positive cells cGAS was found to regulate the levels of the phosphorylated non-homologous end-joining kinase, DNA-PK, which may contribute to H2AX phosphorylation along with other factors. Importantly cGAS was also responsible for increased levels of DNA breaks along with enhanced apoptosis in HPV positive cells but not in HFKs. This study identifies an important and novel role for cGAS in mediating the response of HPV positive cells to chemotherapeutic drugs.

## Author summary

Persistent infection by human papillomaviruses (HPV) is the major risk factor for development of cervical as well as other anogenital and oropharyngeal cancers. Innate immune surveillance pathways are important in determining whether HPV infections will be cleared or persist. The role and activity of cGAS, an innate immune DNA sensor, during HPV infection is still not well understood. In this study we characterized the activity of cGAS-STING pathway in cells that stably maintain high-risk HPV episomes and found it was fully active. Furthermore, our studies indicate that cGAS helps regulate the response to DNA damage causing drugs such as etoposide and cisplatin. Treatment with both drugs further increased the levels of cGAS in HPV positive cells and this was critical for causing DNA breaks along with apoptotic cell death. These findings identify a novel role of cGAS in HPV positive cells in regulating the response to chemotherapeutic DNA damaging agents.

## Introduction

Human papillomaviruses (HPVs) are the etiological agents of benign lesions as well as anogenital and oropharyngeal cancers. Infection by high-risk HPVs is responsible for about 99% of cervical cancers [1] along with approximately 70% of oropharyngeal carcinomas which have increased significantly in numbers in recent years [2,3]. Over two-thirds of adults become infected with high-risk HPVs in their lifetimes, and most will clear the virus within 1 to 2 years. A small number of individuals, however, develop persistent infections that evade immune surveillance and have the potential to progress to cancers. Persistent infection by high-risk HPVs is the greatest risk factor for progression to cancer and the mechanisms used to evade clearance are not well understood [4].

HPVs infect cells in the basal layer of stratified squamous epithelia that become exposed through micro-abrasions. Following entry, HPV genomes are established in the nucleus as extrachromosomal circular DNAs or episomes at 50 to 100 copies per cell. In infected basal cells, early viral genes are expressed and HPV episomes are replicated in S-phase together with cellular chromosomes. Following division, one of the daughter cells leaves the basal layer and begins to differentiate as it migrates to suprabasal layers. Normal cells exit the cell cycle as they leave the basal layer, however, HPV positive cells remain active and re-enter S/G2 in suprabasal layers for productive replication or amplification of viral episomes [5]. Late gene expression is activated coordinately with amplification resulting in synthesis of capsid proteins, assembly of infectious virions and subsequent release to the environment. In cancers, viral genomes are often found integrated into host chromosomes and no virions are produced.

In order to establish persistent infections, HPV proteins must manipulate the host defense mechanisms to avoid immune recognition. The key viral players in this process are the E6 and E7 oncoproteins that bind to and alter the activities of host factors such as p53 and Rb among others [6–9]. These interactions lead to activation of DNA repair mechanisms, altered cell growth control along with the evasion of the innate immune response. Central to immune evasion are the effects of HPV proteins on pattern recognition receptors (PRRs) which are the first line of defense against pathogens [10–12]. Studies have shown that HPV16 E6 induces the degradation of TRIM25 resulting in inhibition of the dsRNA sensor RIG-I [13]. Similarly the activity of another dsRNA sensor, PKR, which regulates protein translation, is impaired by viral proteins [14]. HPVs also target the double strand DNA sensors such as IFI16 which can restrict HPV replication [15] and the AIM2 inflammasome [16]. Another major sensor of dsDNA is the cyclic GMP-AMP synthase (cGAS) but only a limited number of studies have examined its activities in HPV positive cells.

cGAS recognizes and binds cytoplasmic DNA which activates a series of downstream effectors leading to expression of type I interferons (IFN) and interferon stimulated genes (ISGs). Upon binding dsDNA, cGAS synthesizes cyclic GMP-AMP (cGAMP) which is a secondary messenger that binds to the adaptor protein stimulator of interferon genes (STING) [17–20]. Initial binding of cGAMP to STING occurs in the endoplasmic reticulum after which the complex migrates to the Golgi where it recruits the TBK1 kinase inducing STING phosphorylation and TBK1 autophosphorylation. Activated STING in association with TBK1 then phosphorylates the transcription factor IRF3 which translocates to the nucleus to directly activate expression of type I IFN (IFN-I) and ISGs [18,21–24]. Both DNA and RNA viruses have evolved mechanisms to suppress or evade the cGAS-STING pathway to allow for successful infection [25]. The HPV E7 protein has been reported to bind to transiently overexpressed STING and thus inhibit its ability to activate IFN-I synthesis, though other mechanisms could also be responsible for this inhibition [26]. The E7 protein has also been proposed to induce epigenetic silencing of STING and cGAS expression [27]. cGAS often impairs initial viral infections, however, studies using HPV pseudovirions showed a minimal activation of this pathway during entry which is likely due to sequestration of viral DNAs into vesicles that the prevents detection of viral genomes [28]. Overall, it remains unclear how the cGAS-STING pathway functions during persistent HPV infections in physiological relevant human keratinocytes that maintain complete viral episomes.

In addition to recognizing dsDNA from pathogens, cGAS also recognizes damage-associated molecular patterns (DAMPs) or “self” DNAs that have leaked into the cytoplasm after DNA damage and incomplete repair. The DAMP-cGAS pathway also activates the STING-TBK1-IRF3 signaling cascade to induce IFN expression [29]. cGAS is often localized to the cytoplasm but can also be found in the nucleus or in micronuclei that form as a result of genomic instability or DNA damage [30]. In addition, nuclear cGAS has been reported to inhibit the homologous recombination arm of DNA repair [31]. These observations suggest an association of cGAS activation and DNA damage repair mechanisms.

High-risk HPVs activate the ataxia telangiectasia-mutated (ATM) and ATM and Rad3-related (ATR) DNA damage repair (DDR) pathways and this is necessary for viral replication [32]. E6 and E7 proteins activate the ATM and ATR kinases which then phosphorylate Chk2 and Chk1 along with their downstream effectors and this is necessary for viral amplification [33]. HPV proteins activate DDR pathways by inducing increased levels of DNA breaks in both cellular and viral DNAs through the action of cellular factors such as type II topoisomerases [34]. Furthermore, DDR factors are preferentially recruited to viral episomes to mediate the rapid repair of HPV DNAs leading to viral amplification [35–37].

In this study we examined the role of the canonical (STING dependent) and non-canonical functions of cGAS in human keratinocytes that stably maintain viral epsiomes and mimic persistently infected cells in vivo. Our work indicates that cGAS levels are increased in cells that stably maintain HPV episomes and that this occurs at the level of transcription through effects on p53. In contrast to previous reports, the cGAS-STING pathway in HPV positive cells was found to be responsive to cytoplasmic DNA and efficiently activated STING and IRF3. Upon exposure of HPV positive cells to DNA damaging agents, cGAS levels were further elevated in a STING and IFN-I independent manner. This further resulted in enhanced levels of DNA breaks along with increased γH2AX, cleaved caspase 3/7 and apoptosis. Overall, this study identifies a novel association of the cGAS and DDR pathways in HPV positive cells in response to DNA damaging agents.

## Results

### High-risk HPVs modulate cGAS but not STING expression

**Fig 1:**
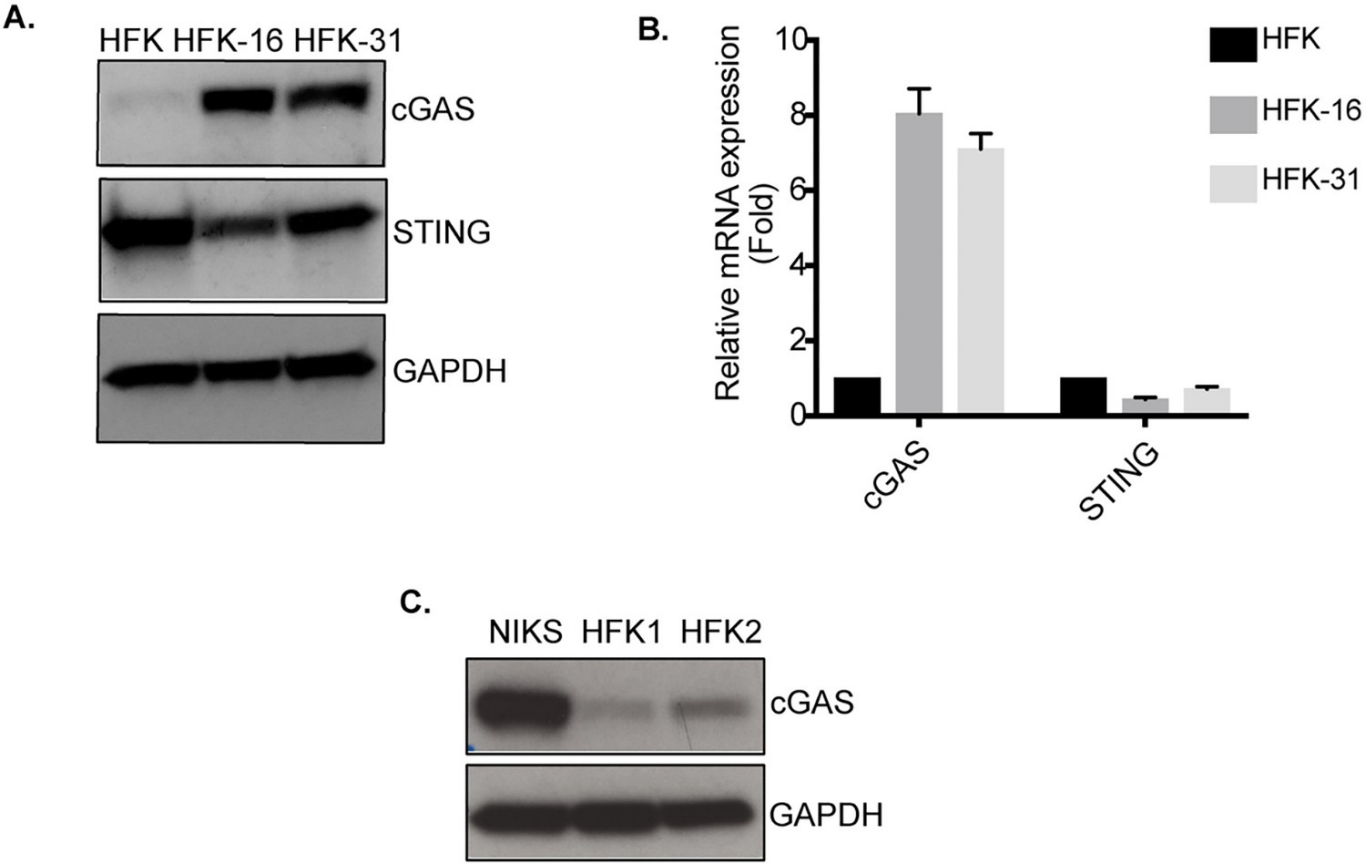
High risk HPVs upregulate cGAS levels at the level of transcription. **(A)** Western blot analysis of cGAS and STING protein levels in primary keratinocytes HFKs along with HPV16 (HFK16) and HPV31 (HFK31) positive keratinocytes. **(B)** Quantitative PCR analysis of mRNA levels of cGAS and STING expressed in HFKs, HFK16 and HFK31 cells normalized to GAPDH as a control. **(C)** Western blot analysis comparing cGAS protein levels between spontaneously immortalized keratinocytes NIKS and primary HFKs from two different donors. Data shown is representative of three independent experiments.

The cGAS-STING pathway plays a critical role in activation of IFN-I expression in response to pathogens or DNA damage. To investigate what role this pathway played in HPV pathogenesis, we first compared the levels of cGAS in human foreskin keratinocytes (HFKs) to cells that stably maintain HPV16 or 31 episomes. HFK-16 and HFK-31 are stable cell lines that were generated by transfection of HFKs with recircularized viral genomes [38]. The levels of cGAS proteins were found to be elevated by up to 7-fold in both HPV 16 and 31 positive keratinocytes as compared to normal HFKs. This increase in cGAS was also observed in mRNA levels as detected by qPCR, indicating that HPV regulates expression at the level of transcription (Fig 1A and 1B). In contrast, minimal differences in levels of STING protein levels were detected between HPV positive and normal keratinocytes. Previous studies have compared cGAS levels in NIKS, a spontaneously transformed human keratinocyte cell line [39], to NIKS with HPV genomes and reported a reduction in cGAS levels in cells expressing viral proteins [27]. We examined the constitutive levels of cGAS in NIKs in comparison to HFKs and observed significantly higher levels in NIKS when compared to primary keratinocytes (Fig 1C). This suggests that use of this immortal keratinocyte cell line may not accurately reflect physiologically significant effects of innate immune signaling by HPVs. Overall this data demonstrates that human keratinocytes that stably maintain high-risk HPVs expressed increased levels of cGAS which was mediated at the level of transcription.

### cGAMP treatment of HPV positive cells results in STING and IRF3 phosphorylation and signaling to IFN-I production

**Fig 2:**
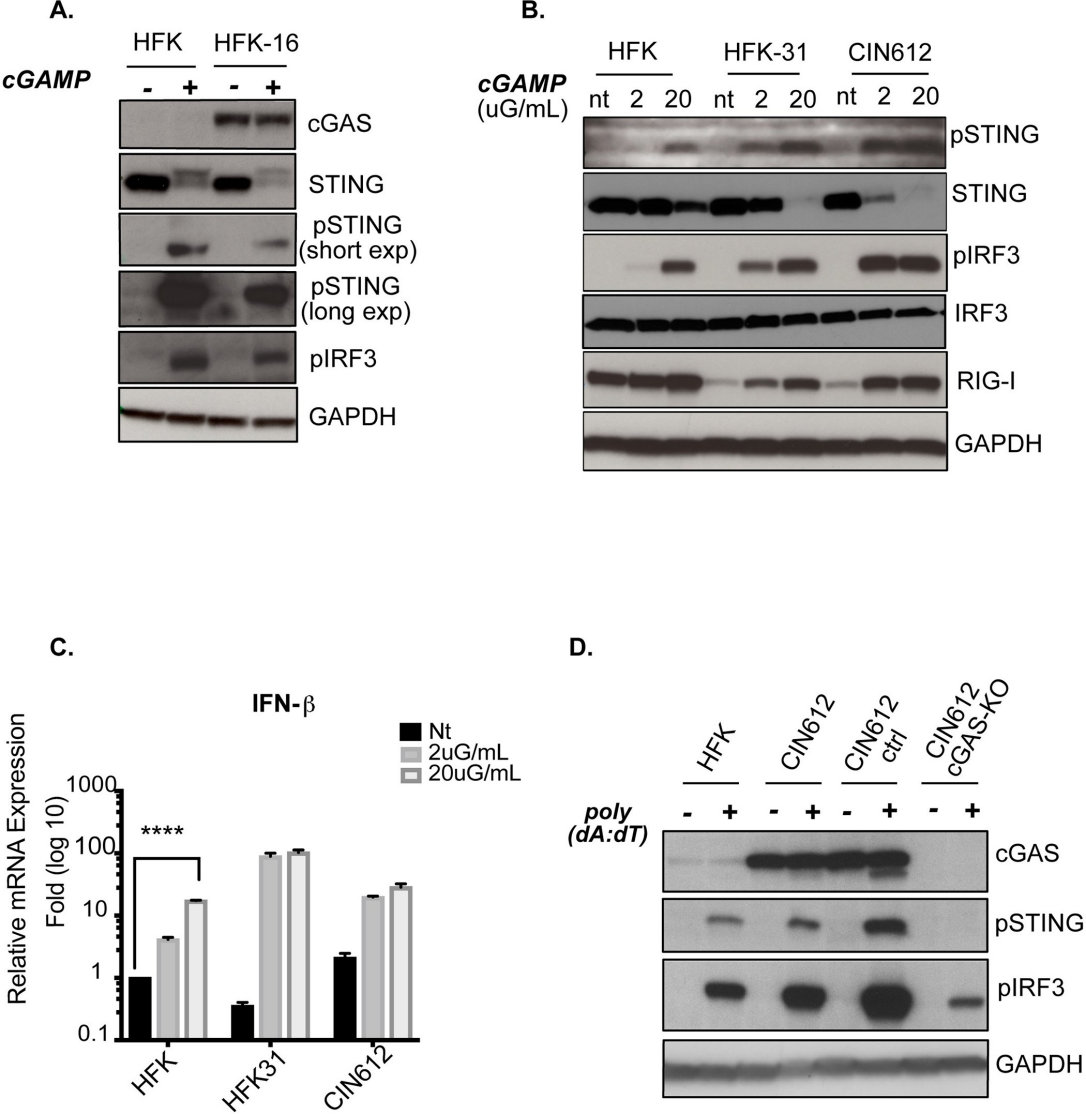
cGAS-STING canonical pathway is active and responsive in high risk HPV positive keratinocytes. **(A)** Western blot analysis of the canonical cGAS-STING factors upon 2’3’-cGAMP treatment. Primary keratinocytes HFKs and HFK16 cells keratinocytes were permeabilized with digitoinin and treated with 5μG/mL cGAMP for 4hrs **(B)** Response of downstream factors in cGAS pathway to different concentrations of cGAMP. HFKs, HFK-31 and CIN612 cells derived from a CIN lesion were examined. Cells were digitonin permeabilized and treated with 2 or 20μG/Ll cGAMP for 4hrs followed by western analysis. **(C)** Quantitative PCR analysis for IFN-β mRNA following cGAMP treatment as described above. Each set includes 3 technical replicates. Levels are shown relative to untreated HFKs. To determine significance of IFN production in HFKs statistical analysis used: two-tailed unpaired T-test; p<0.05 indicates significance compared to control HFK. Where (*) <0.05 and (****) <0.0001. Unpaired T-test to determine significance in HFKs. **(D)** Western analysis of HFK and CIN612 cells for phosphorylated STING and IRF3 at 4hrs post transfection of 1uG/mL poly (dA:dT).

The above studies indicated that levels of cGAS but not STING were increased in HPV positive cells and it was next important to determine if the canonical cGAS-STING pathway was active. For this analysis we examined HFK-31 and HFK-16 cells along with the CIN612 cell line that was derived from a biopsy of a CIN lesion and also maintains HPV genomes as episomes [40] (Fig 2). To determine if the activity of the cGAS canonical pathway was altered in HPV positive cells, keratinocytes were treated with 2’3’-cGAMP, the secondary messenger which acts as a ligand to activate STING. Two different concentrations of cGAMP were used to treat both HPV31 positive cell lines as well as HFKs and then screened for their effects on levels of pSTING and pIRF3. At 4 hours after cGAMP treatment we observed phosphorylation of STING as well as IRF3 in both HPV31 and CIN612 to levels higher than observed with normal keratinocytes (Fig 2B). In addition, HPV positive keratinocytes responded to the lower concentrations of cGAMP to activate both pSTING and pIRF3 (2μG/ml) than seen with HFKs. These observations demonstrate that HPV proteins do not inhibit activation of STING and IRF3 but rather do so more efficiently than HFKs at lower cGAMP concentrations. Next, we quantitated IFN-I expression in response to cGAMP treatment using qPCR. HPV positive cells were found to induce higher levels of IFN expression than seen with HFKs (Fig 2C). A significant increase of approximately 20-fold in IFN-β mRNA levels was observed for HFKs while a greater than 100-fold increase was detected in HPV31 positive cell lines. These results demonstrate that the canonical cGAS pathway is responsive to cGAMP in HPV infected cells, and that STING efficiently signals through IRF3 to induce IFN-I expression.

### cGAS is necessary for cytoplasmic DNA sensing in keratinocytes

In addition to cGAS a number of other intracellular DNA sensors of innate immunity such as IFI16, DAI, AIM2, DDX41, detect aberrantly localized DNAs (reviewed in [41]). To confirm that the effects we were observing were specific to cGAS, we used the CRISPR-Cas9 system to generate stable cGAS knockouts (KO) in the CIN612 cell line along with control cells that contain the empty pLentiCRISPR plasmid and Cas9. The control cells retain expression of cGAS at levels similar to parental CIN612 cells. We generated two independent pooled cultures along with three independently derived clonal lines and found similar results in subsequent experiments with all lines. In the following studies only the data for one cell line will be shown unless specified. Both cGAS-KO and pLenti control CIN612 cells were next analyzed for activation of the cGAS pathway after transient transfection of synthetic dsDNA poly(dA:dT) (Figs 2D and S1). Phosphorylated STING was detected at 4 hours post transfection of dA:dT in HFKs, parental CIN612 and pLenti control cell while no pSTING was detected in cGAS-KO keratinocytes. Furthermore, pIRF3 was detected in all the cells that expressed cGAS but this was greatly reduced in the knockout cells. IRF3 can also be phosphorylated by other DNA sensors such as IFI16, DDX41 (reviewed in [41]), and this may account for the low level, residual activation we observed in HPV positive cells lacking cGAS (Fig 2D). To further confirm activation of cGAS we also measured intracellular cGAMP levels post poly(dA:dT) transfection (S1 Fig) using ELISA assays which showed cGAMP was produced in response to dsDNA in HPV positive cells. These results indicated that cGAS is a functional sensor of cytoplasmic DNA in HPV positive keratinocytes and that signaling occurs through STING to IRF3.

### E6 oncoprotein induces cGAS expression in human keratinocytes

**Fig 3:**
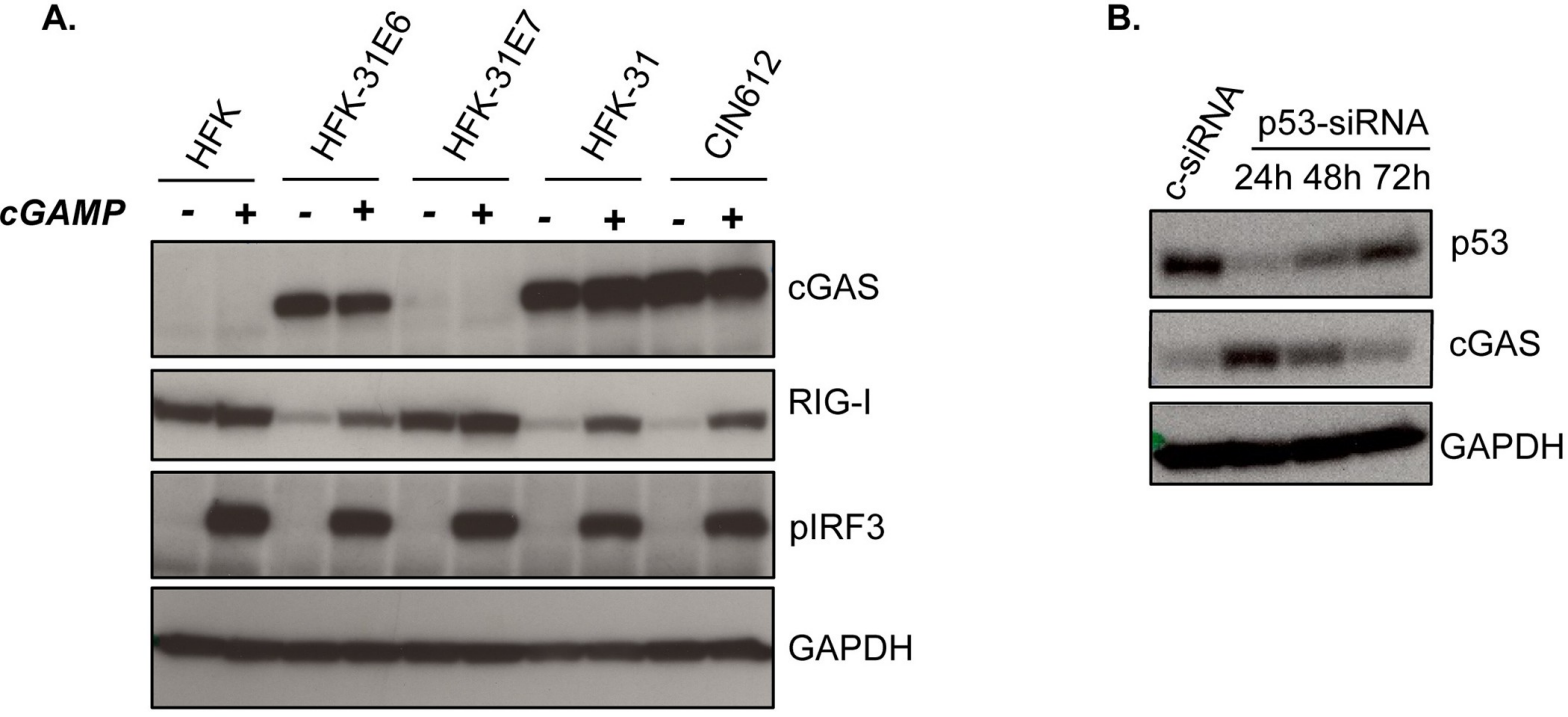
E6 is responsible for increased cGAS expression. **(A)** Western analysis of HFK, HFK-HPV31E6, HFK- HPV31E7, HFK- HPV31E6/E7 and HFK31 cells for factors in canonical cGAS pathway activity in response to digitonin permeabilization and treatment with 5μG/mL 2’3’cGAMP for 4hrs. **(B)** Time course examining cGAS protein levels in HFK-HPV31E7 cells following p53 knockdown at 1 to 3 days post p53 siRNA transfection.

The E6 and E7 oncoproteins provide critical functions in the viral life cycle as well as host cell immortalization. To determine if these oncoproteins were responsible for the increase in cGAS levels, HFKs were transduced with retroviruses expressing HPV31 E6 and/or E7 and stable cell lines were generated. Keratinocytes expressing HPV31 E6 or E6 and E7 displayed high levels of cGAS compared to HFKs or those expressing only E7. In our studies, expression of E6 alone was sufficient to increase cGAS levels indicating that the presence of viral episomes or expression of E7 alone was not responsible. Furthermore, in E6 expressing cells, pIRF3 was induced in response to cGAMP treatment (Fig 3A). While E7 stabilizes p53 proteins, E6 binds and targets p53 for degradation (reviewed in [42]). To determine if the effect of E6 on cGAS expression was dependent on p53, we depleted p53 levels in E7 expressing keratinocytes with transiently transfected siRNAs and performed a time course looking at cGAS levels. siRNA knockdown of p53 initially increased cGAS levels in these cells but at subsequent times (days 2 and 3), as levels of p53 were restored, we observed a corresponding decrease in cGAS expression (Fig 3B). To determine whether this effect was due to p53 transcriptional activation we treated E7 expressing cells with the p53 transactivation inhibitor, pifithrin-α [43], and similar to p53 depletion by siRNA, inhibition of p53 with pifithrin-α resulted in increased levels of cGAS (S2 Fig). This indicates that targeting of p53 by E6 was primarily responsible for the increased levels of cGAS that we observed in HPV positive cells.

### cGAS depletion only modestly impacts episome maintenance and amplification

**Fig 4:**
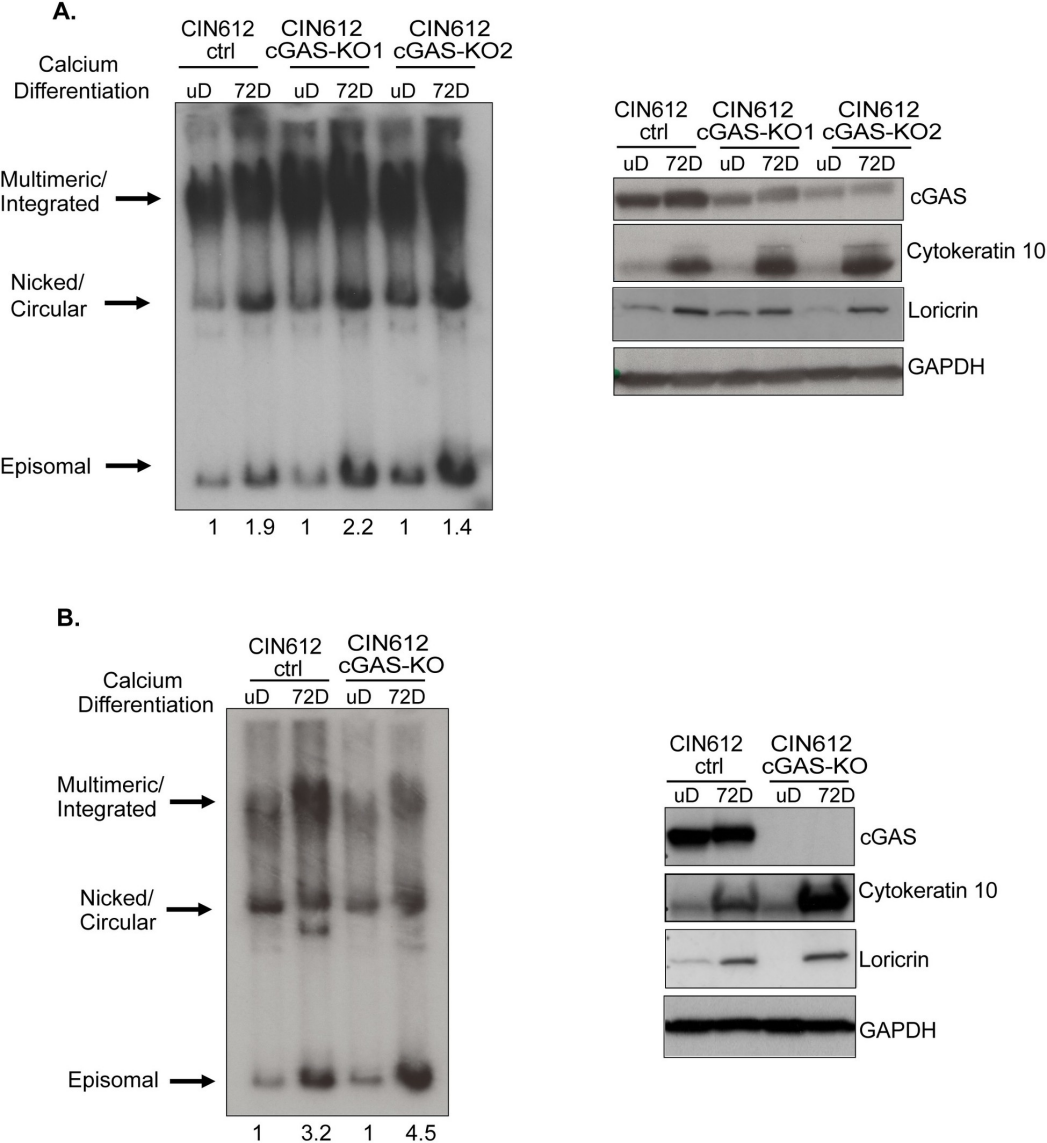
Lack of cGAS has minimal effects on episome maintenance and amplification. **(A)** Southern blot analysis (left) of CIN612 pLenti control pooled cultures and two cGAS-KO pooled cultures. Cells were examined as undifferentiated monolayer cultures or at 72 hours post calcium induced differentiation. Western blot analysis (right) for levels of cGAS and differentiation markers cytokeratin 10 and loricrin. **(B)** Southern (left) and western blot (right) analyses of clonal CIN612 pLenti control and cGAS-KO cell lines. Densitometry values of episomes in both both (A) and (B) were calculated using ImageJ.

Following HPV infection of keratinocytes, viral genomes are established as low copy episomes that can be stably maintained and undergo amplification upon differentiation. To determine if the increased cGAS expression we observed was important for HPV replication, we compared the levels of viral DNA in pLenti control and cGAS-KO cells by southern blot analysis (Fig 4). Two pooled cultures of cGAS knockdown cells (Fig 4A) as well as one clonal knockout line (Fig 4B) are shown. For this analysis we examined cells that were maintained as undifferentiated monolayer cultures as well as differentiation induced at 72 hours following a switch to high calcium media. Differentiation was confirmed by screening for expression of differentiation markers cytokeratin 10 and loricrin while no effect was observed on cGAS expression. Comparing viral genome levels in pLenti control and cGAS-KO cells, only a modest change was observed upon amplification in the absence of cGAS. We also investigated the expression levels of early and late viral gene transcripts in these cGAS knockout cell lines, in either undifferentiated monolayer cultures or following calcium induced differentiation. Quantitative (q)PCR analysis of transcripts from E7 ORF that overlap the late promoter, or E4 and E5 early viral genes are shown (S3 Fig) and no differences were detected between control or cGAS-KO cells.

### DNA damage induces cGAS expression in an IFN-I independent manner

**Fig 5:**
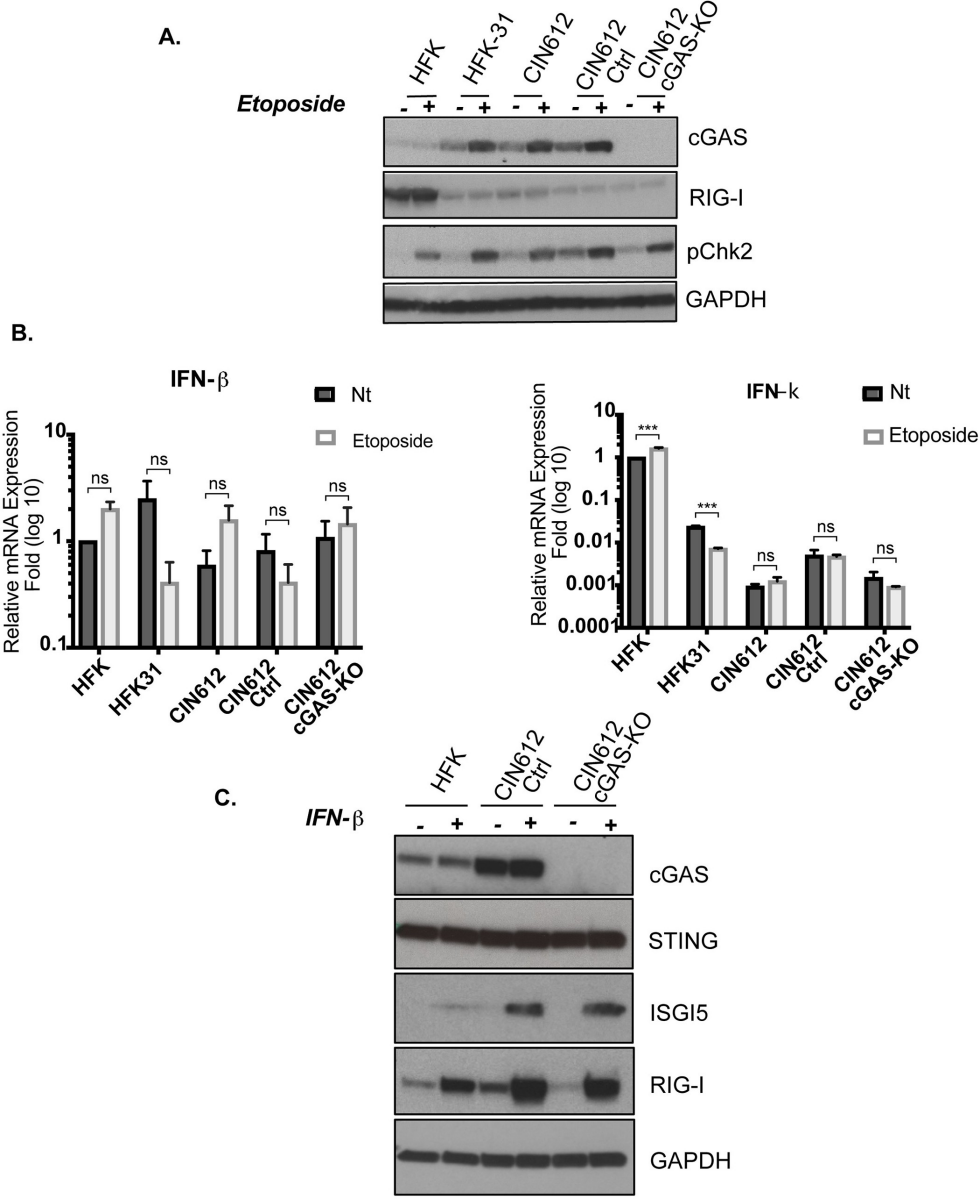
DNA damage induces cGAS expression in HPV positive cells while IFN has no effect. (A) Western blot analysis of cGAS, RIG-I, and pCHK2 of HFKs, HFK-31, CIN612 parental, CIN612 pLenti control and CIN612 cGAS-KO cells following etoposide treatment. Cells were treated with 50uM etoposide for 16hrs prior to analysis. **(B)** qPCR analysis of IFN-β, IFN-κ mRNA levels in same cells upon etoposide treatment. Levels of expression are relative to that in untreated HFKs. **(C)** Effect of IFN-β treatment on levels of cGAS along with two known ISGs, ISG15 and RIG-I. Cells were treated for 16hrs with 200 IU/mL of IFN-β prior to analysis. All data representative of 2 or more independent experiments. Statistical analysis: two-tailed unpaired T-test, where p<0.05 indicates significance (***) <0.001 and ns indicates not significant.

HPVs have been shown to constitutively activate the ATM and ATR DNA pathways and exposure of HPV positive cells to chemotherapeutic drugs further enhances this effect [37]. We next investigated whether treatment of HPV positive cells with DNA damaging agents would also increase cGAS levels. For this analysis we used etoposide, a DNA topoisomerase II inhibitor that introduces DNA double strand breaks [44], to treat HFKs, pLenti controls and cGAS-KO cells. Consistent with previous reports, the levels of pChk2, a downstream effector of DDR pathways, were increased in both HPV positive and HFKs following etoposide treatment. Importantly, the levels of cGAS increased in HPV31 positive cells following treatment but not in normal HFKs or cGAS-KO cells (Fig 5A). Furthermore, while levels of cGAS transcripts increased upon etoposide treatment, no change was observed with E6 mRNAs (S4 Fig). DNA damage has been reported to activate the interferon response and some studies have suggested that cGAS is an ISG [45]. To determine whether the observed increase in cGAS levels was due to activation of interferons, we quantitated IFN-I production following etoposide treatment. In addition to IFN-β, keratinocytes express another interferon, IFN kappa (κ), which is constitutively expressed in keratinocytes, but is repressed in HPV positive cells [46,47]. Using qPCR we determined that etoposide treatment did not significantly elevate levels of IFN-β or κ in HPV positive cells (Fig 5B). Next, we examined the levels of the RNA sensor RIG-I, as a representative ISG, for any interferon effects due to etoposide treatment. HPVs downregulates RIG-I [13] and we did not detect any effect of etoposide on this sensor. To further confirm that the observed modulation of cGAS was not due to IFNs, normal and HPV31 keratinocytes were treated with 200 units of human IFN-β and this failed to increase cGAS levels but did induce the expression of ISGs such as ISG15 or RIG-I (Fig 5C). Finally, to exclude the`possibility we were missing and early transient response to etoposide we also quantitated IFN-β mRNA levels at 1 to 8 hours post drug treatment (S5 Fig) but no effect was observed. Overall, these studies indicate that DNA damage induced by etoposide increased cGAS protein levels in HPV positive cells but not HFKs and that this occurs independent of IFN-I induction.

### cGAS localizes in HPV induced micronuclei

**Fig 6:**
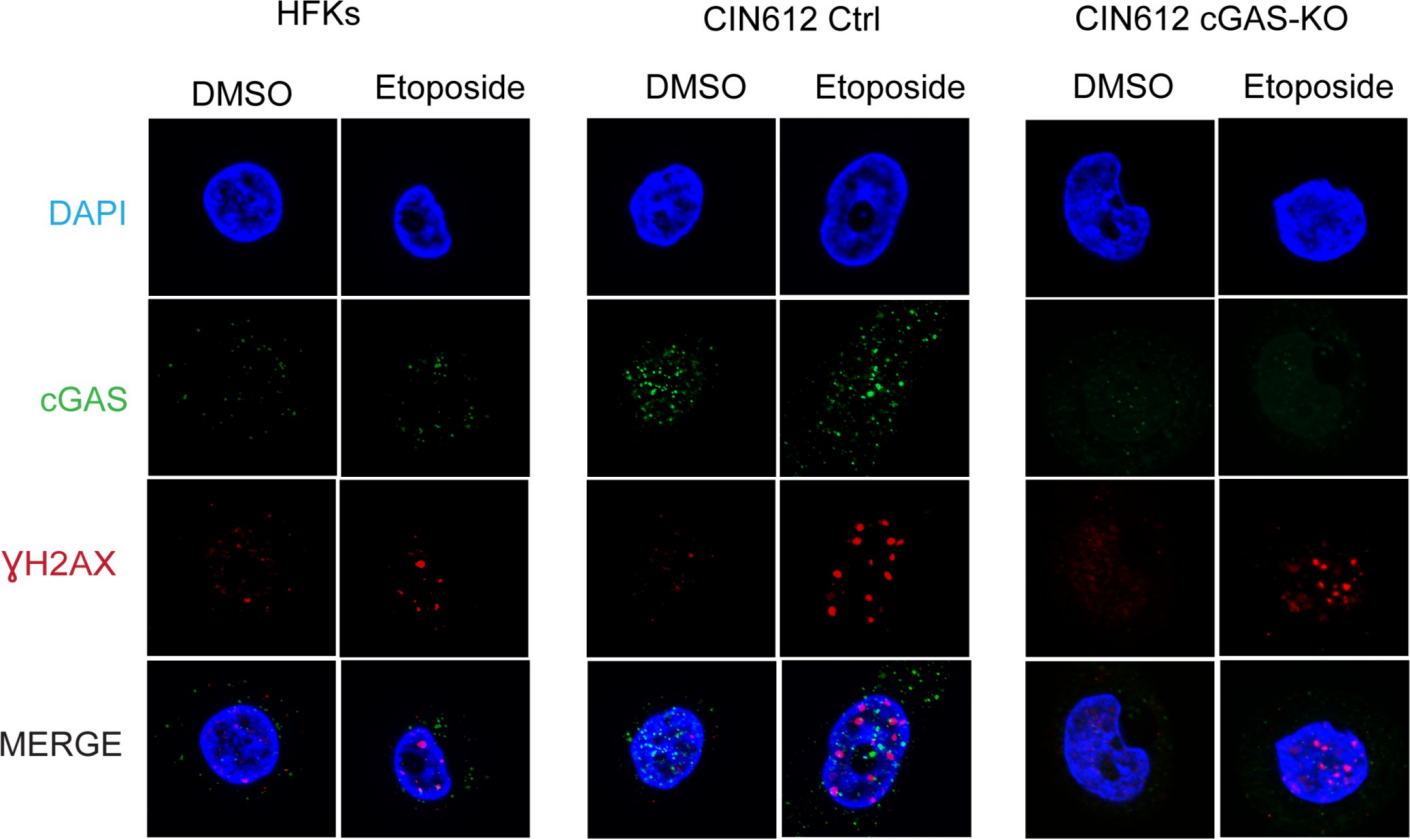
cGAS localization in keratinocytes upon DNA damage induction. Immunofluorescence analysis for cGAS and γH2AX proteins in HFKs, CIN612 pLenti controls, and CIN612 cGAS-KO keratinocytes following treatment with 50μM etoposide for 24hrs.

**Fig 7:**
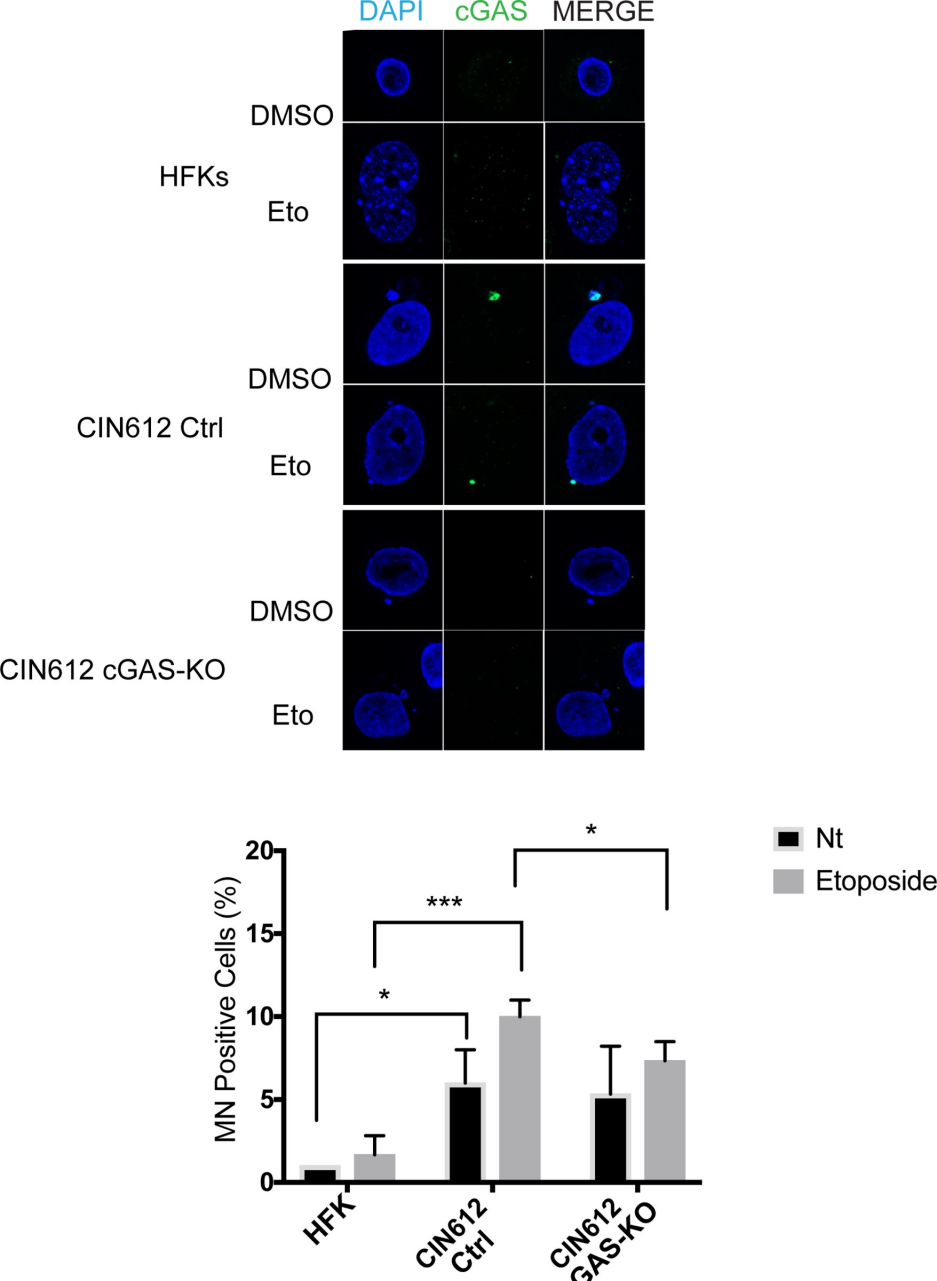
cGAS localizes in DNA damage induced micronuclei. Micronuclei formation in HPV positive cells and cGAS co-localization (left panel) post etoposide treatment for 24hrs at 50μM. Quantitation of micronuclei from three independent experiments and two different clones of CIN612 pLenti control or cGAS-KO (right panel). Analysis was performed on 100 cells for each condition. Statistical analysis: two-tailed unpaired T-test; p<0.05 indicates significance compared to the respective sample group. Where (*) <0.05 and (***) <0.001.

cGAS was initially characterized as a cytoplasmic DNA sensor but recent studies indicate it can also be found in the nucleus under conditions of genotoxic stress [31]. Moreover, cGAS has been detected in micronuclei which are extra-nuclear structures that form as a result of chromosome fragmentation due to genomic instability and DNA damage [30]. Clinical studies have suggested a correlation between advanced stages of CIN and the frequency of micronuclei formation [48, 49]. To determine where cGAS is localized in HPV positive cells, we performed immunofluorescence analysis of monolayer cultures of HPV31 positive keratinocytes and normal keratinocytes in the presence or absence of DNA damaging agents. In HPV positive cells, cGAS was localized to punctate-like structures as well as in micronuclei while in HFKs the signal was more broadly distributed, less intense and micronuclei were rarely observed. Treatment of HPV positive cells with etoposide resulted in a further increase in the intensity and frequency of cGAS puncta along with enhanced numbers of cGAS positive micronuclei. No such increases were observed in treated HFKs (Fig 6). γH2AX foci also were observed but no significant overlap with cGAS was detected (Fig 6). Next, we quantitated the frequency of micronuclei in untreated pLenti control cells and noted that the knockout cells did not substantially alter the number of micronuclei observed. In contrast, the frequency of micronuclei increased about two-fold in pLenti control CIN612 cells following treatment with etoposide but not in cGAS-KOs. Importantly, all the micronuclei detected in either treated or untreated pLenti control CIN612 keratinocytes were cGAS positive (Fig 7). This indicates that cGAS moderately regulates levels of micronuclei formation post etoposide treatment but since they were still detected in cGAS-KO cells it was not essential for their formation during HPV induced genomic instability. The cGAS signal in the micronucleus in CIN 612 ctrl cells is strong and it hides the signal from cGAS in cytosolic and nuclear foci as well as in another micronucleus. A higher exposure of this panel is shown in S7 Fig showing the cGAS foci and second micronucleus staining along with magnifications of the two micronuclei.

### Loss of cGAS reduces levels of γH2AX and cleaved caspases 3 and 7

**Fig 8:**
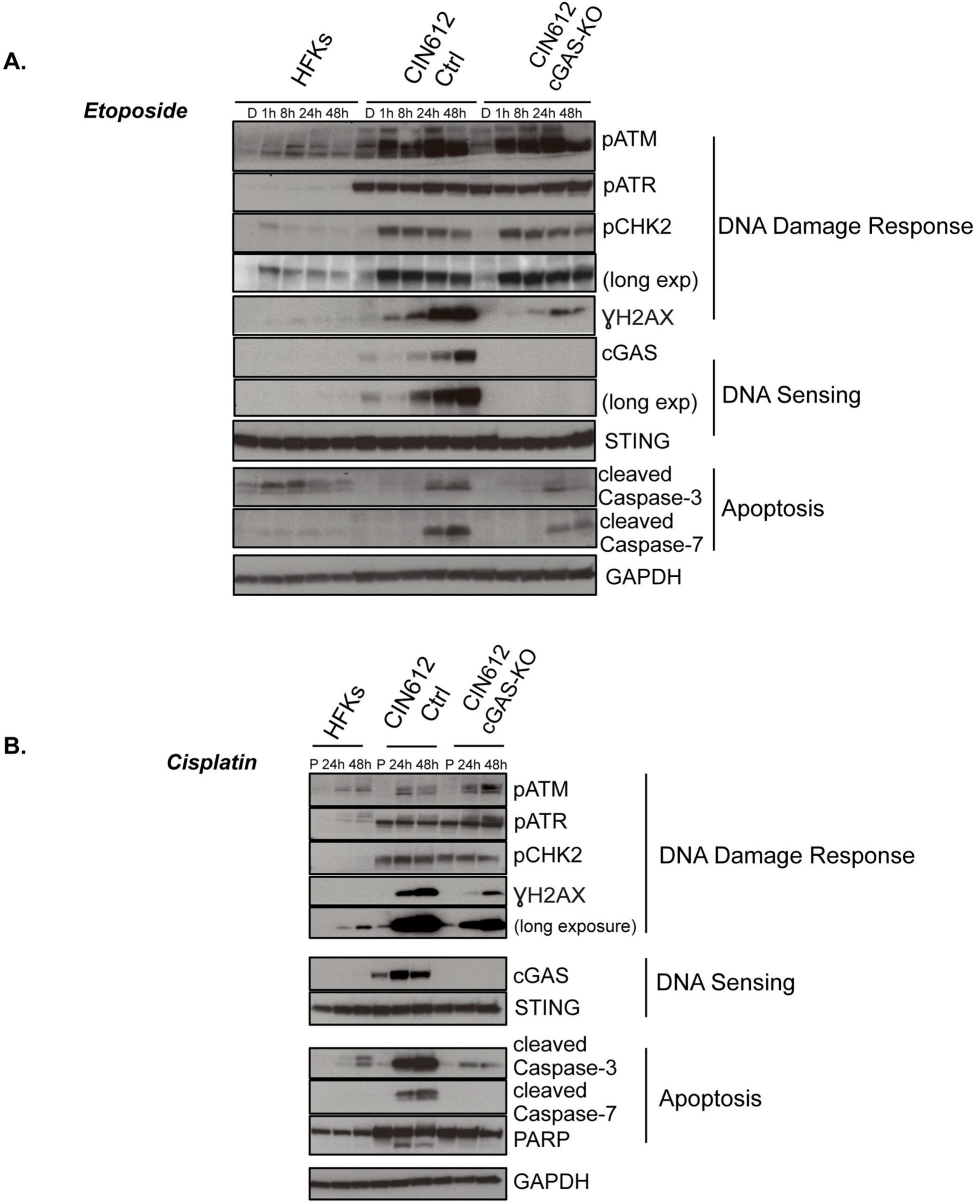
HPV positive cells lacking cGAS display lower levels of γH2AX and cleaved caspases 3/7. **(A)** Time course western blot analysis for DNA damage repair factors and cleaved caspase 3/7 in CIN612 pLenti control and CIN612 cGAS-KO cells in response to 50 μM etoposide treatment or DMSO for the indicated times. **(B)** Time course western analysis of same cells as in (A) in response to 5μM cisplatin treatment or PBS for 24 and 48 hrs. Similar results were seen in three or more independent experiments.

We next sought to investigate whether cGAS affected activation of DDR pathways by screening for effects on downstream effectors. For this analysis we first examined untreated cells as well as following treatment with etoposide. Consistent with previous reports [50], untreated pLenti CIN612 control cells as well as cGAS-KO exhibited higher levels of pATM and pATR than HFKs. Upon exposure to etoposide, the levels of pATM and pCHK2 increased substantially while those of pATR were only minimally enhanced. Interestingly, the levels of cGAS increased in control CIN612 cells but not in HFKs or cGAS-KO. In contrast, the levels of γH2AX, a surrogate marker for DNA breaks, increased following treatment of pLenti control cells but were substantially reduced in cGAS-KO cells (Fig 8A). Since treatment with etoposide can induce apoptosis, we next screened for the levels of two apoptotic proteases, cleaved caspase 3 and 7, and found both to be significantly reduced in the absence of cGAS. This indicates that cGAS is responsible for enhanced levels of γH2AX along with cleaved caspase 3/7 in HPV positive cells following exposure to etoposide.

To determine whether the effects of cGAS were specific to etoposide, we treated cells with cisplatin a drug that is often used for treatment of head and neck cancers (Fig 8B). Cisplatin is a platinum ion based drug that binds and induces DNA lesions leading to ATR activation along with apoptotic cell death [51]. Cisplatin treatment also increased levels of cGAS expression as well as pATR. Furthermore, reductions in levels of γH2AX as well as cleaved caspase 3/7 and PARP1 were seen in cells lacking cGAS and is consistent with the etoposide studies. This indicates that cGAS is a critical regulator of the response of HPV positive cells to chemotherapeutic drugs.

### DNA-PK and H2AX phosphorylation in HPV positive cells

**Fig 9:**
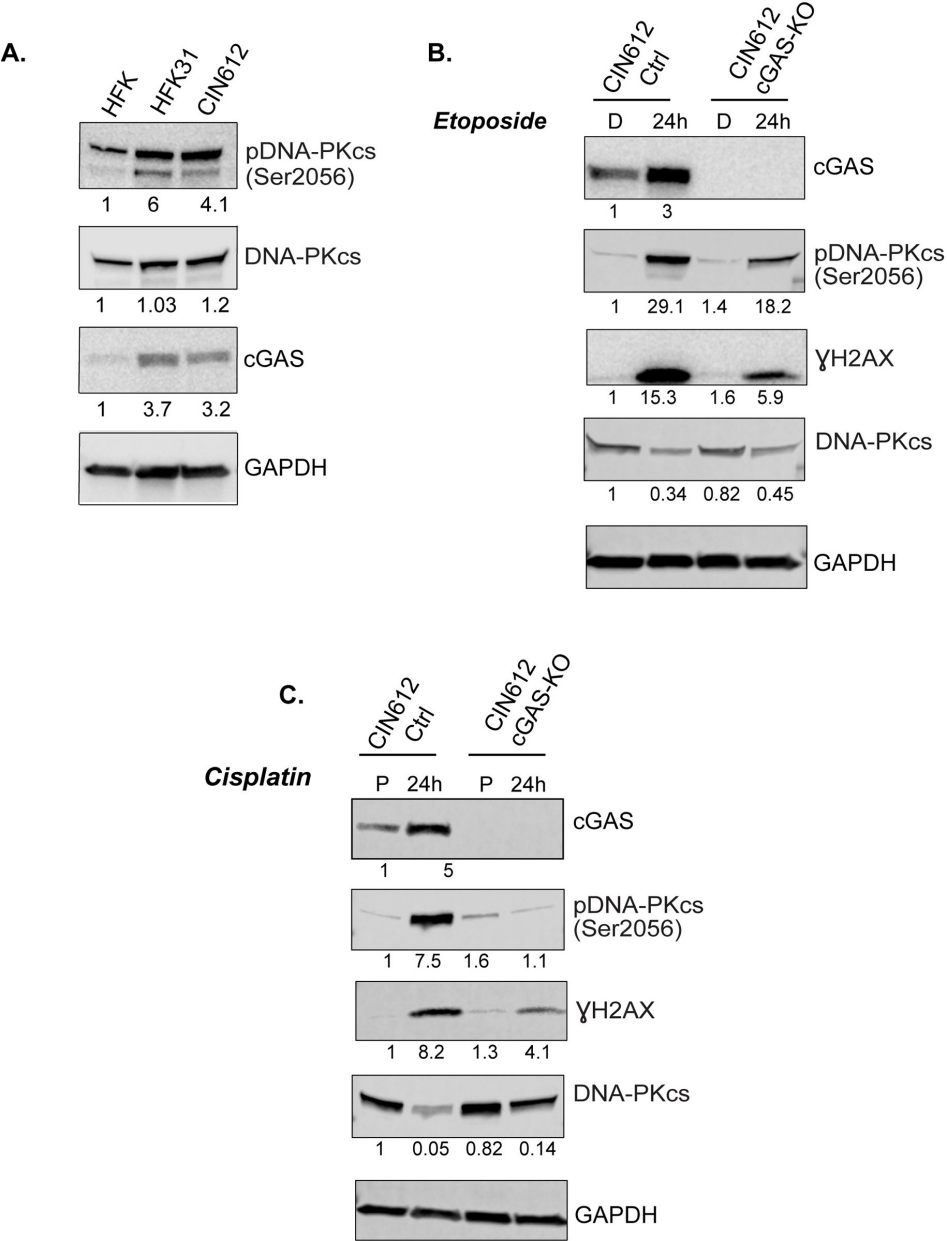
pDNA-PK levels are decreased in absence of cGAS. **(A)** Western blot analysis of total and phosphorylated DNA-PKcs levels in HFKs, HFK-31 and CIN612 cells. CIN612 pLenti control and cGAS-KO cells were screened for levels of cGAS, DNA-PKc, pDNA-PKc Ser2056 and γH2AX following treatment with etoposide (50μM) **(B)** and cisplatin (5μM) **(C)** for 24hrs. Densitometry values were determined using ImageJ.

We next investigated what factors might be involved in cGAS dependent phosphorylation of H2AX in response to treatment with DNA damaging agents. Three kinases phosphorylate H2AX: pATM, pATR and pDNA-PK. Our studies examining changes in pATM or pATR levels in response to etoposide or cisplatin treatment showed minimal effects upon cGAS knockout. In contrast to ATM and ATR that repair damaged DNA through homologous recombination, DNA-PK is involved in non-homologous end joining (NHEJ) [52]. Interestingly, we observed that HPV positive cells maintain higher levels of pDNA-PKcs as compared to normal keratinocytes (Fig 9A). DNA-PK has been shown to mediate phosphorylation of H2AX following high levels of DNA fragmentation or upon apoptosis [53]. In our studies, analysis on the levels of phosphorylated DNA-PKcs following treatment of wildtype and cGAS knockout cells with DNA damaging drugs demonstrated substantially reduced levels in cells lacking cGAS (Fig 9B and 9C). This indicates that cGAS regulates pDNA-PKcs levels in HPV positive cells in response to DNA damaging agents and may be linked to regulation of γH2AX as suggested in previous studies examining the effects of radiation and staurosporine [53].

### cGAS inhibits DNA damage repair

**Fig 10:**
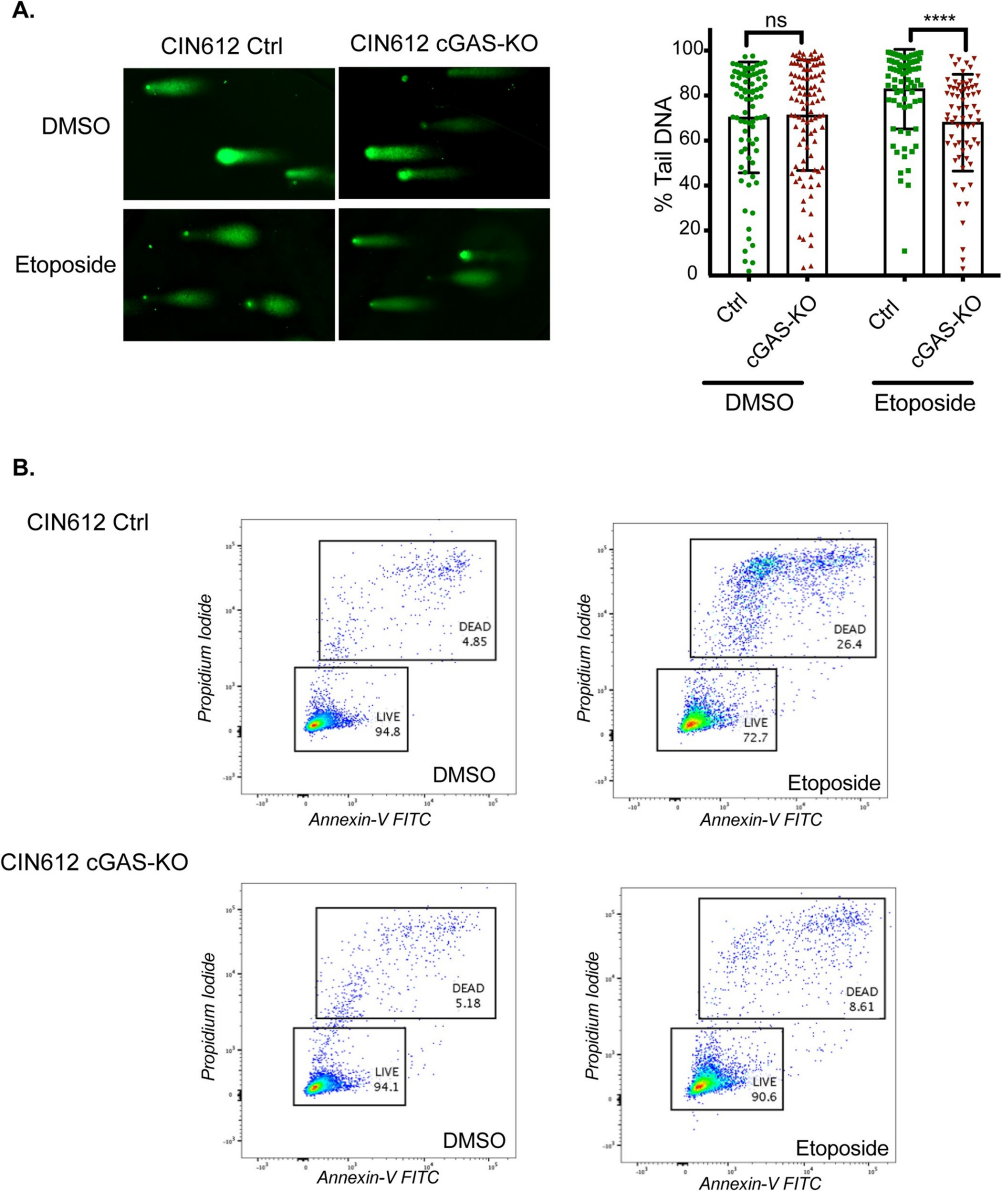
cGAS promotes DNA damage induced apoptosis of HPV positive cells. (A) Levels of DNA breaks in CIN612 pLenti control and cGAS-KO cells as determined by alkaline COMET assays at 2 hrs following etoposide treatment. Representative images of comets are shown in right panel and quantitation of comet percent tail DNA from three independent experiments including 2 different clones of pLenti control and 2 clones of cGAS-KO cells are shown in left panel. Tail length was calculated using OpenComet software for FIJI. Statistical analysis: two-tailed unpaired T-test, where p<0.05 indicates significance and ns indicates not significant. **(B)** Flow cytometric analysis of apoptotic cells as measured by Annexin V and PI staining at 24hrs post 10μM etoposide treatment. Graphical analysis plotted and quantitated using FlowJo software. Similar results were seen in three independent experiments.

The above studies indicated that high levels of cGAS lead to enhanced amounts of γH2AX which often correlates with DNA breaks. We therefore investigated if DNA break formation was dependent on cGAS using alkaline comet assays with comet tail length representative of the amount of breaks. It has been previously reported that HPV keratinocytes contain high levels of DNA breaks in comparison to normal cells [37]. We quantitated comet tail lengths of pLenti and cGAS-KO cells before or after etoposide treatment (Fig 10A) and did not observe any significant difference in levels in untreated cells. In contrast, treatment with etoposide induced substantially higher levels of DNA breaks in pLenti control cells as compared to cells lacking cGAS which exhibited no changes. These results indicate that cGAS is a critical regulator of DNA break formation in HPV positive cells in response to DNA damaging agents.

### Depletion of cGAS inhibits DNA damage induced apoptosis of HPV infected keratinocytes

Treatment of cells with chemotherapeutic agents such as etoposide and cisplatin induces DNA breaks which can lead to apoptosis. We next analyzed cell viability by flow cytometry using annexin-V as a marker of early apoptosis and propidium iodide (PI) to distinguish late apoptosis from necrosis. In the experiment shown in Fig 10B, after 24 hours treatment with etoposide approximately 21% cell death increase over untreated controls was observed in pLenti controls as compared to 3.4% for cGAS knockout cells. Similar results were observed in three independent experiments. Altogether these results demonstrate that in HPV infected cells cGAS provides a pro-apoptotic function following treatment with DNA damaging agents.

## Discussion

cGAS activates expression of interferon through the STING/TBK1/IRF3 pathway in response to cytoplasmic DNA from pathogens or following DNA damage. Our studies identified a critical function of cGAS in HPV pathogenesis in human keratinocytes that stably maintain HPV episomes and mimic persistently infected cells in vivo. The levels of cGAS were found to be significantly increased in HPV 16 and 31 positive cells in comparison to normal human keratinocytes and this increase occurred at the level of transcription. No differences were, however, observed in levels of STING or IRF3. Furthermore, the cGAS-STING pathway was fully active in response to exogenously added cGAMP as well as poly (dA:dT) leading to cGAMP synthesis and phosphorylation of STING as well as IRF3. These observations contrast with previous reports using the spontaneously immortalized keratinocyte line, NIKS [39], that suggested that cGAS levels were suppressed in HPV positive NIKS cells [27]. Our studies indicate cGAS expression is increased by over 6-fold in NIKS in comparison to normal human keratinocytes indicating they may not accurately recapitulate physiological activities. It has also been reported that E7 can bind to transiently overexpressed STING and this leads to inhibition of IFN expression [26]. Our studies show that STING is efficiently phosphorylated and active in HPV31 and HPV16 positive cells leading to IRF3 phosphorylation. This demonstrates that the caonical pathway is fully functional in HPV positive cells and that other activities of E7 are likely responsible for inhibition of interferon expression. One such activity is E7 mediated impairment of STAT-1 transcription that results in inhibition of IFN expression. Impaired expression of STAT-1 by E6 and E7 is critical for HPV replication as studies showed that restoration of STAT-1 to levels seen in HFKs blocked viral replication [54]. We conclude that cGAS levels are increased in human keratinocytes that stably maintain HPV episomes and that the STING/TBK1/IRF3 pathway is fully functional in these cells.

The increase in cGAS levels was found to be due to the action of E6 through effects on p53. We transiently knocked down p53 with siRNAs in keratinocytes expressing E7 alone and observed corresponding increases in cGAS levels. Similarly, treatment with an inhibitor of p53 transcriptional activity also resulted in increased cGAS expression. These studies identified a novel function of p53 as a transcriptional repressor of cGAS expression. While cGAS is often regulated at the level of protein stability, HSV infection of neonates also leads to increased levels of cGAS transcription and similar increases are seen in vivo in tumors such as non-small-cell lung cancers [55, 56]. A role for p53 in regulating cGAS has, however, not been previously described. In our studies, treatment of HPV positive cells with cGAMP induced higher amounts of pSTING and pIRF3 than seen in HFKs as well as responsive to lower amounts of cGAMP. This suggested that a form of “priming” may occur in HPV positive cells due to unrepaired DNA damage. Expression of either E6 or E7 has been shown to induce high levels of DNA breaks that leads to fragmented DNAs leaking into the cytoplasm. While both viral proteins can induce DNA breaks, only E6 was shown to increase cGAS levels indicating that in addition to DNA damage, impairment of p53 is also required. cGAS transcription is regulated by factors such as Sp1 and CREB [57] and p53 could act through these factors or instead act directly on the promoter. cGAS has also been reported to be an ISG that is responsive to IFNs but addition of exogenous IFN-β failed to increase its expression in HPV positive cells. The increased levels of cGAS found in HPV positive cells suggested it may provide an important function in the viral life cycle such as modulating replication. Knockdown of cGAS with CRISPR methods, however, had a minimal effect on episome maintenance as well as calcium induced differentiation-dependent amplification indicating it functioned in other aspects of the HPV life cycle. Furthermore, knockdown of cGAS had no effect on viral transcription and while we cannot exclude the possibility it might affect translation, no such activity has previously been demonstrated for cGAS.

Since high-risk HPVs constitutively activate DDR pathways and cGAS has been linked to DNA damage repair mechanisms, we investigated if cGAS impacted this process in keratinocytes persistently infected with HPV. We first investigated where cGAS was localized in these cells that maintain viral episomes. Recognition of cytoplasmic dsDNA by cGAS involves it’s binding to two dsDNA molecules in a 2:2 oligomeric complex that form liquidlike droplets puncta in the cytoplasm [58]. Following genotoxic stress or DNA damage cGAS also translocates to the nucleus where it has been reported to form complexes with γH2AX, disrupt the PARP1-Timeless complex and inhibit repair [31]. Additional studies have suggested it impairs RAD51’s ability to form D-loops which are critical in mediating homologous recombination DNA repair [59]. cGAS can also localize to micronuclei that are formed upon DNA damage and contain fragmented DNAs [30]. In our studies cGAS was found localized to puncta in both the cytoplasm and the nuclei as well as in micronuclei that are present at high levels in HPV positive cells but rarely in HFKs. Furthermore, all micronuclei in HPV cells were cGAS positive and in cells containing micronuclei cGAS was predominantly concentrated in these structures. Lack of cGAS expression did not reduce the frequency of micronuclei formation in untreated HPV positive cells. In contrast following exposure of cells lacking cGAS to DNA damaging agents the frequency of micronuclei was reduced by about 50% suggesting a contributing role in regulating their formation under these conditions. These observations contrast with studies in U2OS cells [30] which reported that knockdown of cGAS increased the frequency of micronuclei in both untreated or ionizing radiation treated cells indicating the effects we observed may be specific to HPV positive cells. cGAS has also been reported to co-localize with γH2AX in the nucleus [31]. While we observed high levels of γH2AX foci in HPV positive cells, no co-staining in puncta was observed with only a low-level overlap in micronuclei. Treatment of cells with etoposide increased the frequency and intensity of γH2AX puncta as well as those containing cGAS but still no overlap was observed.

Treatment of HPV positive cells with either etoposide or cisplatin increased the levels of cGAS while no such increase was seen in HFKs. Surprisingly, knockdown of cGAS reduced the levels of γH2AX in response to either drug but had minimal effects on pATM, pATR and pChk2. In contrast, cGAS knockout substantially reduced the levels of pDNA-PKcs. DNA-PK has been reported to phosphorylate H2AX under conditions of DNA damage and fragmentation following exposure to staurosporine which coincided with degradation of ATM [53]. DNA-PK is activated by autophosphorylation, which our studies indicate is regulated by cGAS but has a minimal effect on the other DDR kinases. The demonstration of an association of cGAS with pDNA-PK in HPV positive cells is novel and indicates a potential linkage to the non-homologous end joining pathway. Most of the current knowledge links cGAS to HR pathways and little is known about its involvement in NHEJ. Previous studies identified pDNA-PK as the primary kinase responsible for phosphorylating H2AX following treatment with DNA damaging agents such as radiation or staurosporin [53]. Preliminary studies involving treatment of HPV positive cells with the pDNA-PK inhibitor AZD7648 partially inhibited its phosphorylation and only moderately reduced levels of H2AX phosphorylation. This indicates other factors also contribute to phosphorylation of H2AX in HPV positive cells. Investigation of a role for DNA-PK in HPV pathogenesis is an important area for future studies. Previous studies also suggested that cGAS inhibits the homologous recombination repair pathways but our studies show activation of ATM and ATR still occurs at high levels in HPV positive keratinocytes. We cannot exclude the possibility that cGAS is acting on downstream effectors such as RAD51 leading to its re-localization. It has been shown that in conditions of genotoxic stress RAD51 together with RPA retain ssDNA in the nucleus preventing its leakage to the cytoplasm and cGAS activation [60]. Furthermore, Wallace et al. reported that while the levels of homologous recombination factors were increased in HPV positive cells their activity was impaired by about 50% [61]. This impairment was the result of E6 mediated re-localization of RAD51 and it is possible this is due to the action of cGAS.

Etoposide blocks the activities of type II topoisomerases and it was recently shown that these enzymes are responsible for the majority of DNA breaks in HPV positive cells [34]. Examination of the levels of DNA breaks in HPV positive cells by COMET assays in the absence or presence of cGAS failed to show any difference in untreated cells. In contrast, treatment with etoposide, increased the frequency of DNA breaks in cells with wildtype cGAS but not in knockout cells. This further indicates that a primary of function of cGAS in HPV positive cells is in mediating the response to DNA damaging agents. Treated HPV positive cells were also examined for levels of apoptosis by screening for annexin V and PI staining and the lack of cGAS was found to greatly reduce the number of apoptotic cells. This reduction also correlated with reduced levels of cleaved caspase 3 and 7 in cGAS knockout cells. We conclude that HPV positive cells are sensitized to DNA-damage induced apoptosis through the action of cGAS. This identifies an important link between the innate immune sensor cGAS and DNA damage in HPV pathogenesis.

## Materials and methods

### Antibodies

The antibodies used in this study are: cGAS (Cell Signaling, catalog no. 15102); cleaved Caspase 3 (Cell Signaling, catalog no.9664); cleaved Caspase 7 (Cell Signaling, catalog no. 9491); Cytokeratin 10 (Santa Cruz Biotechnology, catalog no. sc-23877); **γ**-H2AX (Millipore, catalog no. 05–636); GAPDH (Santa Cruz Biotechnology, catalog no. sc-47724); IRF3 (Cell Signaling, catalog no.4302); ISG15 (Cell Signaling, catalog no. 2743); Loricrin (BioLegend, catalog no. 905101); p53 (Santa Cruz Biotechnology, catalog no. sc-126); PARP (Cell Signaling catalog no. 9532); phosphorylated ATM (Cell Signaling, catalog no.13050); phosphorylated ATR (Cell Signaling, catalog no. 2853); phosphorylated Chk2 (Cell Signaling, Catalog no. 2661); phosphorylated DNA-PKcs (Ser2056) (Cell Signaling catalog no. 68716); phosphorylated IRF3 (Cell Signaling, catalog no. 4947); phosphorylated STING (Cell Signaling, catalog no. 19781S); phosphorylated STING (Cell Signaling, catalog no. 19781S); RIG-I (Santa Cruz Biotechnology, catalog no. sc-376845); STING (Cell Signaling, catalog no. 13647). Secondary antibodies: anti-mouse IgG, HRP-linked Antibody (Cell Signaling, catalog no. 7076) and anti-rabbit IgG, HRP-linked Antibody (Cell Signaling, catalog no. 7074). Additional antibodies used for immunofluorescence: anti-MB21D1 (Sigma-Aldrich, catalog no. HPA031700); donkey anti-mouse IgG highly cross-secondary antibody, Alexa Fluor 568 conjugate (Invitrogen, catalog no. A10037); donkey anti-rabbit IgG secondary antibody, Alexa Flour 488 conjugate (Invitrogen, catalog no. A21206).

### Cells

Primary keratinocytes (HFKs) were isolated from neonatal human foreskins as previously described [62]. HPV16 and HPV31 cell lines were generated in HFKs by co-transfection of each respective circular viral genome with pSV-Neo2 plasmid [38]. 48 hours post transfection cells were treated with four doses of G418 every other day. Initially HFKs were treated with two doses of 200 μg/ml G418, followed by two 100 μg/ml and fed with Mitomycin C treated NIH-3T3 J2 fibroblasts on the alternate days. Drug resistant keratinocytes were then cultured and passaged in E-media and fed with arrested NIH-3T3 J2.

CRIRSPR Cas9 cGAS knockout cells were generated from CIN612 keratinocytes. Four different sgRNAs targeting human cGAS were selected from a database [63] and cloned into lentiviral transfer plasmids as described by Zhang Lab [64]. Briefly, each pair of primers were phosphorylated, annealed and ligated into LentiCRISPR v2 (gift from Feng Zhang, Addgene plasmid #52961 [65]) digested with BsmBI. The constructs were next transformed into One Shot Stbl3 chemically competent *E*.*Coli* (Invitrogen) and colonies were picked and sequenced for the sgRNA inserts using U6 primer. DNA from the confirmed colonies for cGAS sgRNAs or empty LentiCRISPR v2 plasmid, as a control, were next co-transfected in HEK 293T using Fugene 6 (Promega) together with lentiviral packaging plasmids pCMV-VSV-G (gift from Bob Weinberg Addgene plasmid #8454 [66]) and psPAX2 (gift from Didier Trono, Addgene plasmid #12260). HEK 293T cells were supplemented with fresh media 16 hours post transfection. 24 hours later supernatants were collected and used to transduce CIN612 keratinocytes with 8 μg/ml polybrene (Sigma-Aldrich). After 4 courses of puromycin selection every other day, cell pools were analyzed for cGAS expression by western blotting. Only two of the sgRNAs were successful in knocking out cGAS and used in our studies. These were: sg4 FW 5’-CACCAGCTTCCGCACGGAATGCCAG-3’, Rev 5’-AAACCTGGCATTCCGTGCGGAAGCT-3’ and sg9 FW 5’-CACCAGAATGCCAGGGGCGCCCCGA-3’, Rev 5’-AAACTCGGGGCGCCCCTGGCATTCT-3’. Finally, keratinocyte pools were selected by single cell limited dilution and each clone analyzed by western blotting prior to use.

HFKs stably expressing the viral oncogenes E6 and/or E7 were generated from plasmids pLXSN-31E6, pLXSN-31E7; pLXSN-31E6E7 or empty vector control. Each construct was transfected in PT67 retroviral packaging cell line and viral supernatants were used for transduction of HFKs. Protocol previously described in detail by Mehta and Laimins [37].

Transient siRNA knockdown of p53 was carried in HFK-31E7 cells (described above). Human p53 siRNA (Santa Cruz Biotechnology, sc-29435) or control siRNA (Santa Crus Biotechnology, sc-37007) were transfected with TransIT keratinocyte transfection reagent (MirusBio) in keratinocytes following manufacturer’s protocol. Cell lysates were collected at 24–72 hours post transfection and analyzed by western blotting.

### Drugs

Etoposide and Cisplatin were purchased from Sigma-Aldrich and dissolved in DMSO or PBS respectively. Pifithrin- α was purchased from Sigma-Aldrich and dissolved in DMSO.

### Cell culture

Keratinocytes used in this study (such as HFKs, HFK16, HFK31 and CIN612) were cultured in E-medium supplemented with 5 ng/ml EGF (Epidermal growth factor, mouse natural; Corning, catalog no.354010) and co-cultured with NIH-3T3 J2 fibroblasts. E-medium recipe has have been previously described [62].

NIH-3T3 J2 fibroblasts were cultured and passaged in DMEM (Dulbecco’s Modified Eagle Medium, Gibco) supplemented with 10% newborn calf serum (R&D Systems) and 1% Penicillin-Streptomycin (Gibco). Prior to co-culturing with keratinocytes, fibroblasts were growth arrested by treatment with 8 ng/ml Mitomycin C for 3-4hrs.

HEK 293T cells were cultured in DMEM supplemented with 10% FBS (Fetal bovine serum, Sigma Aldrich) and 1% Pen-Strep.

### Transfection

3 x 10^5^ keratinocytes were seeded in 6 well plates. Next day the cells were transfected with 1μg/well poly(dA:dT) (Invivogen) and TransIT keratinocyte transfection agent (MIrusBio). NIH 3T3 J2 feeder cells were removed prior to the assay using versine solution. Lysates were collected 4 hours post transfection using RIPA buffer and processed for Western Blot as described below. Data shown are representative of 3 independent experiments.

### cGAMP ELISA

Keratinocytes were transfected with poly (dA:dT) as described above. Four hours post transfection, NIH 3T3 J2 feeder cells were removed using versine solution and keratinocytes analyzed for intracellular cGAMP production using manufacturer’s instructions (2’3’–cGAMP ELISA Kit, Cayman Item No. 501700). Data representative of 3 independent experiments.

### cGAMP delivery

Treatment of keratinocytes with 2’3’-cGAMP (Invivogen) was carried in digitonin permeabilization buffer (50mM HEPES (pH7), 100 mM KCl, 3mM MgCl_2_, 0.1 mM DTT, 85mM Sacharose, 1 mM ATP, 0.2% BSA). The cells were permeabilized in the buffer with or without cGAMP for 15min followed by a PBS wash and addition of fresh media. After 4 hours of incubation, lysates were collected to be analyzed for protein and mRNA expression. Data shown are representative of 3 independent experiments.

### Western blot analysis

For all experiments, keratinocytes were initially released of Mitomycin C treated NIH-3T3 J2 by a 2–3 min incubation and 2x washes with phosphate-buffered saline (PBS) with EDTA pH8. Next keratinocytes were lysed in RIPA buffer supplemented with cOmplete Protease Inhibitor Cocktail (Roche) and incubated on ice for 30min. Following incubation lysates were centrifugated at 12,000 x g for 15min at 4^0^ C and supernatants were collected. Protein concentration was determined by Bradford assay and lysates were separated in 4–20% Criterion TGX precast midi protein gel (Bio-Rad). Proteins were transferred to polyvinylidene difluoride (PVDF) membrane and next blocked in 5% milk in TBS-Tween followed by probing for the specific primary at 1:1000 dilution and secondary antibodies at 1:5000 dilution as described above. Immunoblotting for GAPDH was used as a loading control for all experiments.

### Real-time qPCR analysis

Total RNA was isolated using EZ-10 Spin Column kit (BioBasic) and cDNA was generated by High-Capacity RNA-to-cDNA (Invitrogen) following manufactures protocol. Real-time qPCR was performed using LightCycler 480 (Roche) with SybrGreen reagent.

KiCqStatrt primers (Sigma) were used for each gene: cGAS; STING; IFNb; IFNk and GAPDH. Samples were run in triplicates and relative fold change determined using comparative CT method with GAPDH as an endogenous control. Primers used for viral gene expression were as follows; E4 Fw 5’-ACCACATCGAATTCCAAAACC-3’, Rev 5’-TTCTGTGCTCTGGCTCTGTTC-3’; E5 Fw 5’-TTTGCTTTGCTTTTGTGTGCT-3’, Rev 5’-AACAACGTAATGGAGAGGTTGC-3’; E7 Fw 5’-GCGTGGAGAAACACCTACGTT-3’ and Rev 5’-CTCATCTGAGCTGTCGGGTAA-3’. The primers used for E7 overlap the late promoter region.

### Calcium induced cell differentiation

CIN612 cells were differentiated as previously described [62]. Cells were seeded at ~ 5 x 10^6^ in 10cm dishes and 16 hours later switched to M154 media (Life Tech) with 0.03mM CaCl_2._ After 24 hours keratinocytes were switched to M154 with 1.5mM CaCl_2_ and incubated for another 72 hours to differentiate prior to isolation. DNA was purified using Phenol:Chloroform;Isoamyl Alcohol (ThermoFisher). Southern blot analysis was performed as previously described by Mehta and Laimins [37].

### Immunoflorescence

Keratinocytes were plated at 50,000 cells/well on sterile glass coverslips thickness 1 and incubated overnight. Next day cells were treated with etoposide for 24 hours. Following drug treatment, cells were fixed with 4% formaldehyde solution and blocked in normal goat serum (Life Technologies) with primary antibodies overnight at 1:200 dilution, followed by fluorescent secondary antibodies at 1:400 dilution for 1 hour in the dark. Finally, the cells were mounted and DAPI stained with Vectashield hardset antifade mounting medium with DAPI (Vector Laboratories) and visualized with a Zeiss florescence microscope with ApoTome (Carl Zeiss). Images were analyzed using FIJI software.

### Alkaline comet assay

CIN612 keratinocytes were treated with 5uM etoposide or equal volume DMSO for 2 hours. Next, feeder fibroblasts were removed by short incubation following 2x washes of PBS with EDTA. Keratinocytes were then lifted by trypsin-EDTA, centrifuged at 1000g for 5 minutes, resuspended in pre-warmed molten LM Agarose and plated on glass slides. Next the cells were lysed, incubated in alkaline unwinding solution and ran through electrophoresis as per manufactures’ instructions (Trevigen Comet Assay). Finally, the COMETs were visualized using AMG EVOS microscope and OpenComet plugin for FIJI to quantitate % tail DNA. Alkaline comet assay is more sensitive than neutral comet assay as it detects both ss and ds DNA breaks. Data shown is a cumulative of 3 independent experiments including 2 different clones of CIN612 pLenti control and cGAS-KO cells. GraphPad Prism7 was used to perform statistical analysis for two-tailed unpaired t-test, where P<0.05 was considered statistically significant.

### Annexin V Flow Cytometry

To determine the amount of apoptosis upon drug treatment, annexin V was used to label apoptotic cells with Annexin V-FITC Apoptosis Detection Kit (Sigma-Aldrich). Labeled samples were then run in BD FACSCanto II flow cytometer and FlowJo software was used to plot and analyze the data. Data shown representative of three independent experiments.

### Statistical analysis

All statistical analysis and p-values were determined using GraphPad Prism7 Software.

## Supporting information

S1 Fig HPV positive cells synthesize 2’3’ cGAMP. ELISA assay quantitation of intracellular cGAMP levels at 4hrs post poly (dA:dT) transfection. Amounts of cGAMP were determined using the standard curve, as per manufacturer’s instructions. Similar results were seen in at least three independent experiments.
(TIF)

S2 Fig Inhibition of p53 activity modulates cGAS levels. cGAS mRNA levels of HFK-HPV31E7 keratinocytes after treatment with 100uM Pifithrin-alpha (α) for 8 or 24 hrs. cGAS levels were determined by qPCR and expressed relative to DMSO treated cells. Data are representative of three or more independent experiments.
(TIF)

S3 Fig Lack of cGAS does not affect viral gene expression. qPCR for early viral transcripts: E4 and E5, and late: E7 expression; in undifferentiated and calcium induced differentiated keratinocytes. One CIN612 pLENTI control and two CIN612 cGAS-KO clonal cell lines were analyzed. Data representative of two independent experiments.
(TIF)

S4 Fig Etoposide effect on E6 and cGAS mRNA levels. qPCR analysis for expression of E6 and cGAS after treatment with 50uM Etoposide for 16hrs. Fold change expressed relative to DMSO treated cells.
(TIF)

S5 Fig Etoposide does not cause an early interferon response in primary or HPV positive keratinocytes. Cells were treated with DMSO or 50uM Etoposide for the indicated times and IFNβ or cGAS levels were determined by qPCR analysis. Fold change expressed relative to HFK DMSO.
(TIF)

S6 Fig Complete western blots for pSTING post cGAMP treatment. The entire blots for pSTING bands shown in Fig 2A for short and long exposures are presented.
(TIF)

S7 Fig Higher exposure of cGAS in micronuclei and foci. Higher intensity exposure of CIN612 ctrl panel in Fig 6 post etoposide treatment showing nuclear and cytosolic foci along with staining in a second micronucleus. A close-up image of the two micronuclei in this cell is also included.
(TIF)
