# Supplementary figures and images for: Human papillomaviruses sensitize cells to DNA damage induced apoptosis by targeting the innate immune sensor cGAS
Source: PLoS Pathog. 2022 Jul 25;18(7):e1010725. doi: 10.1371/journal.ppat.1010725 (PMC9352202; doi:10.1371/journal.ppat.1010725)

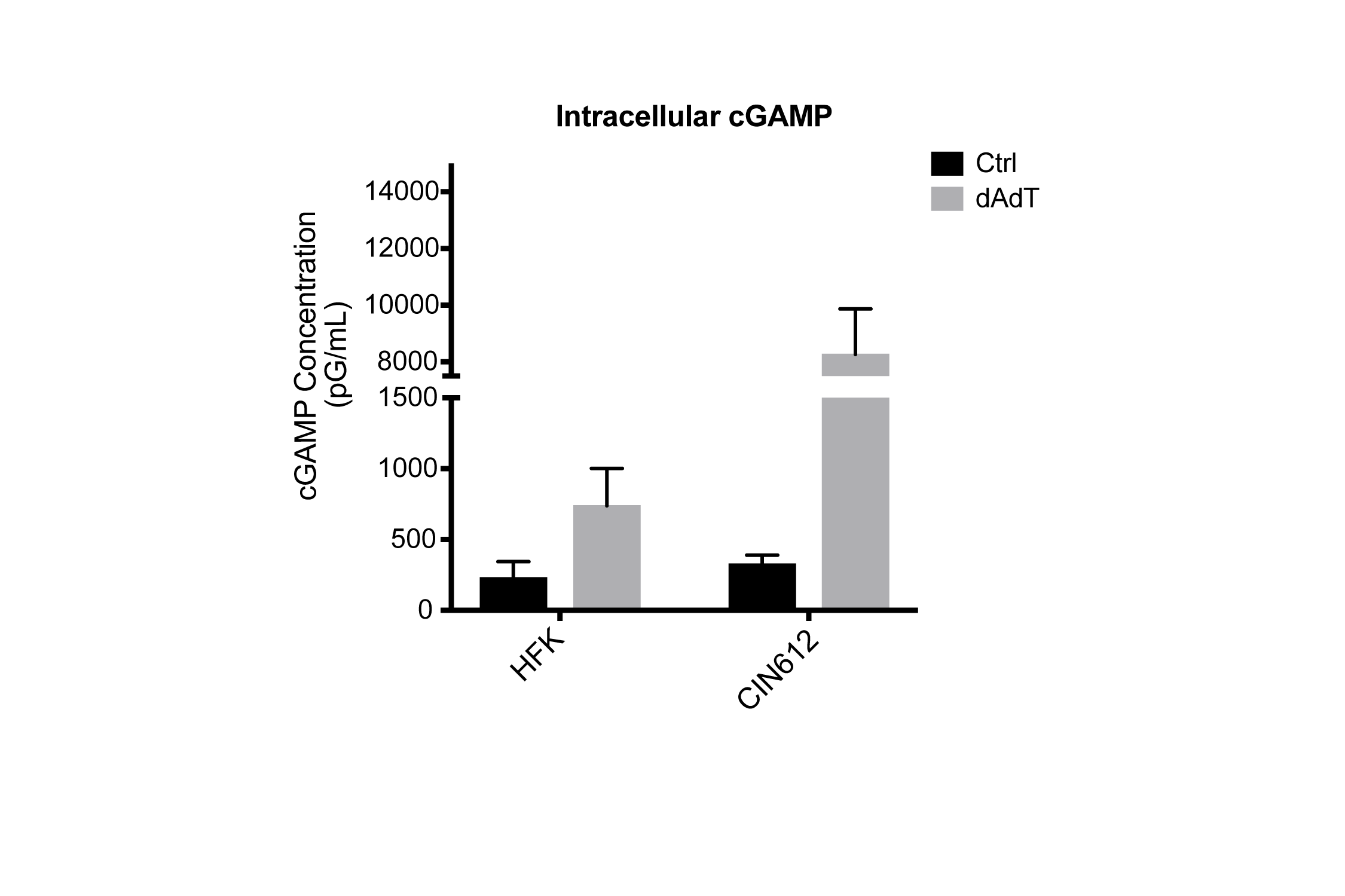

Supplement: S1 Fig — ELISA assay quantitation of intracellular cGAMP levels at 4hrs post poly (dA:dT) transfection. Amounts of cGAMP were determined using the standard curve, as per manufacturer’s instructions. Similar results were seen in at least three independent experiments. (TIF) [file ppat.1010725.s001.tif]

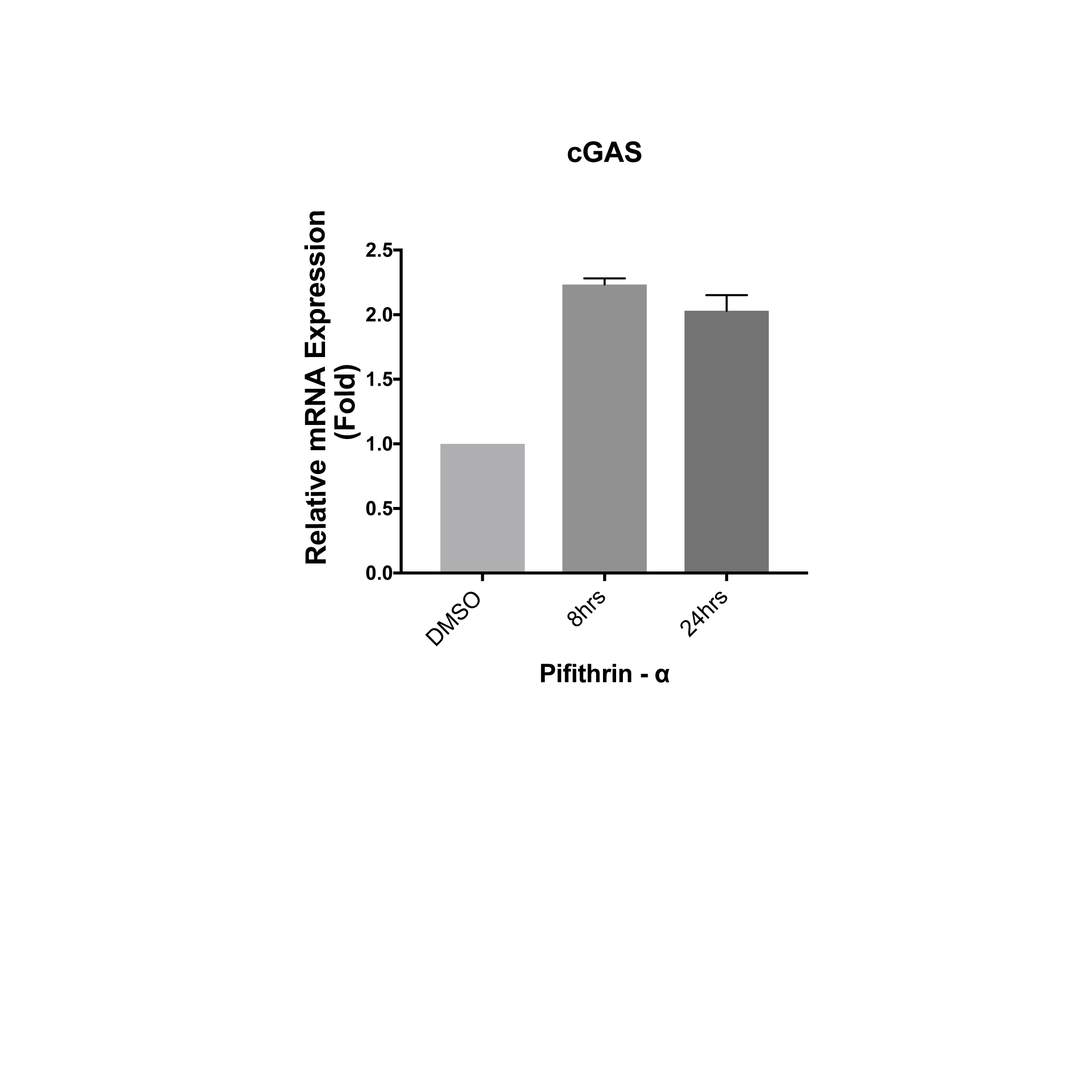

Supplement: S2 Fig — cGAS mRNA levels of HFK-HPV31E7 keratinocytes after treatment with 100uM Pifithrin-alpha (α) for 8 or 24 hrs. cGAS levels were determined by qPCR and expressed relative to DMSO treated cells. Data are representative of three or more independent experiments. (TIF) [file ppat.1010725.s002.tif]

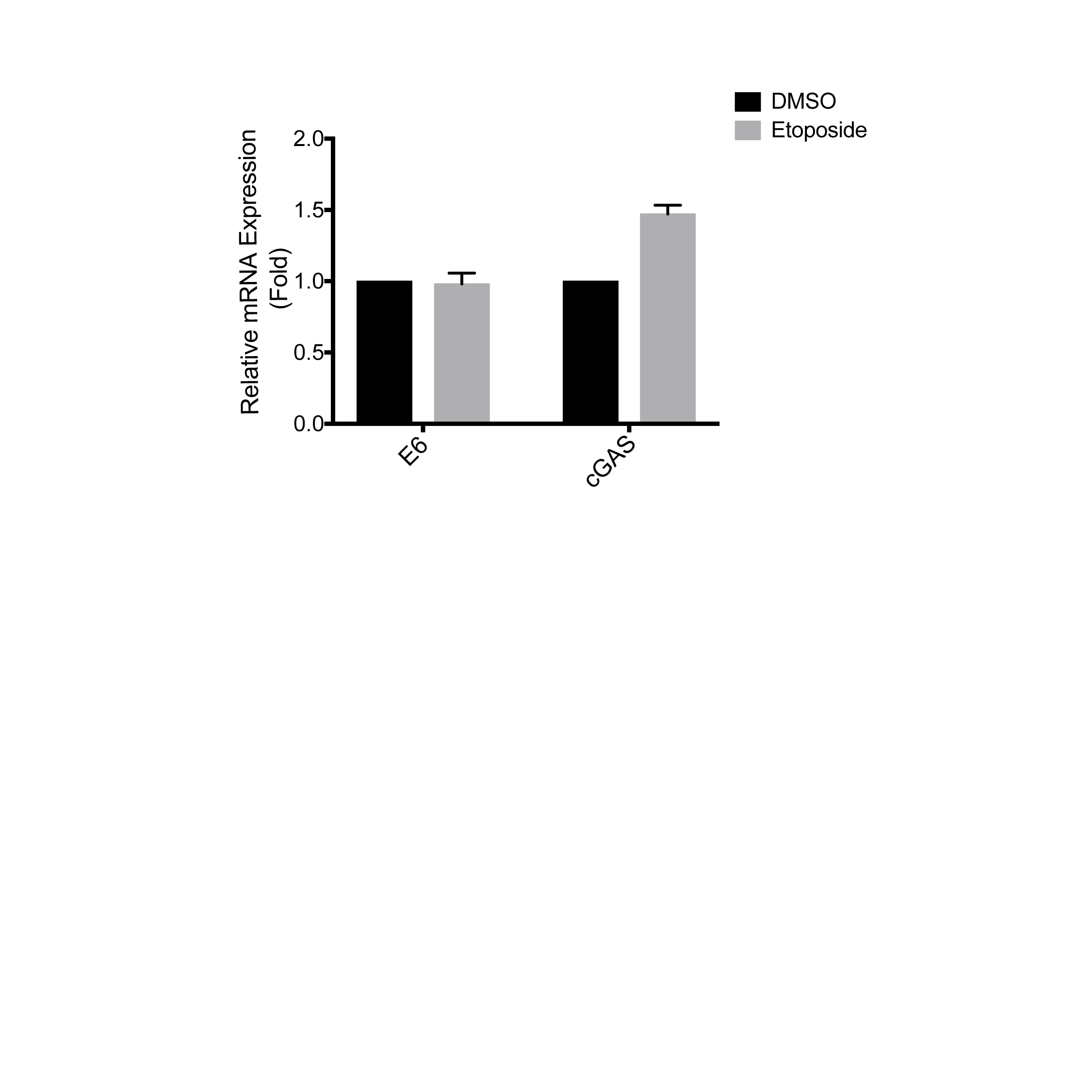

Supplement: S4 Fig — qPCR analysis for expression of E6 and cGAS after treatment with 50uM Etoposide for 16hrs. Fold change expressed relative to DMSO treated cells. (TIF) [file ppat.1010725.s004.tif]

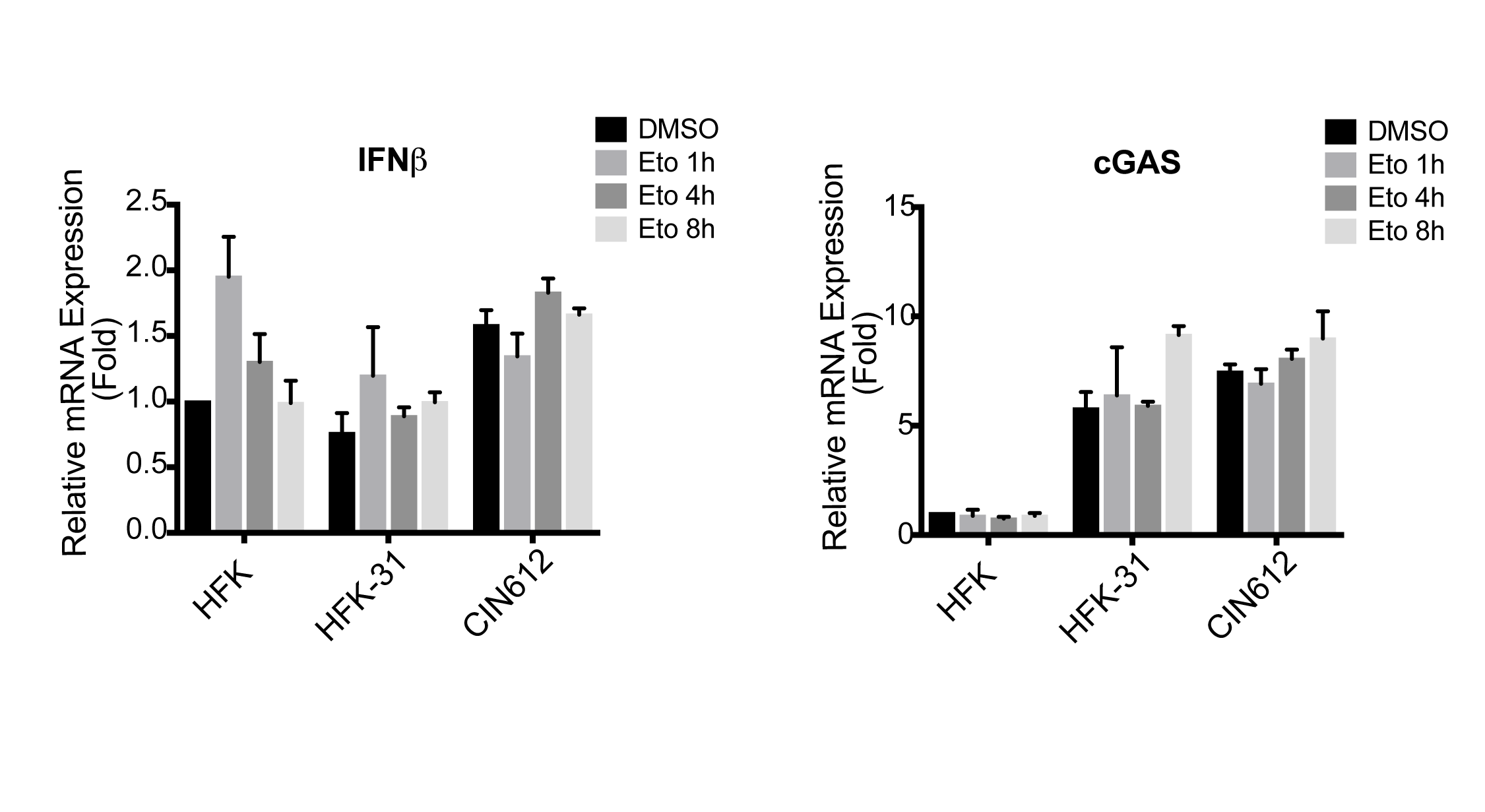

Supplement: S5 Fig — Cells were treated with DMSO or 50uM Etoposide for the indicated times and IFNβ or cGAS levels were determined by qPCR analysis. Fold change expressed relative to HFK DMSO. (TIF) [file ppat.1010725.s005.tif]

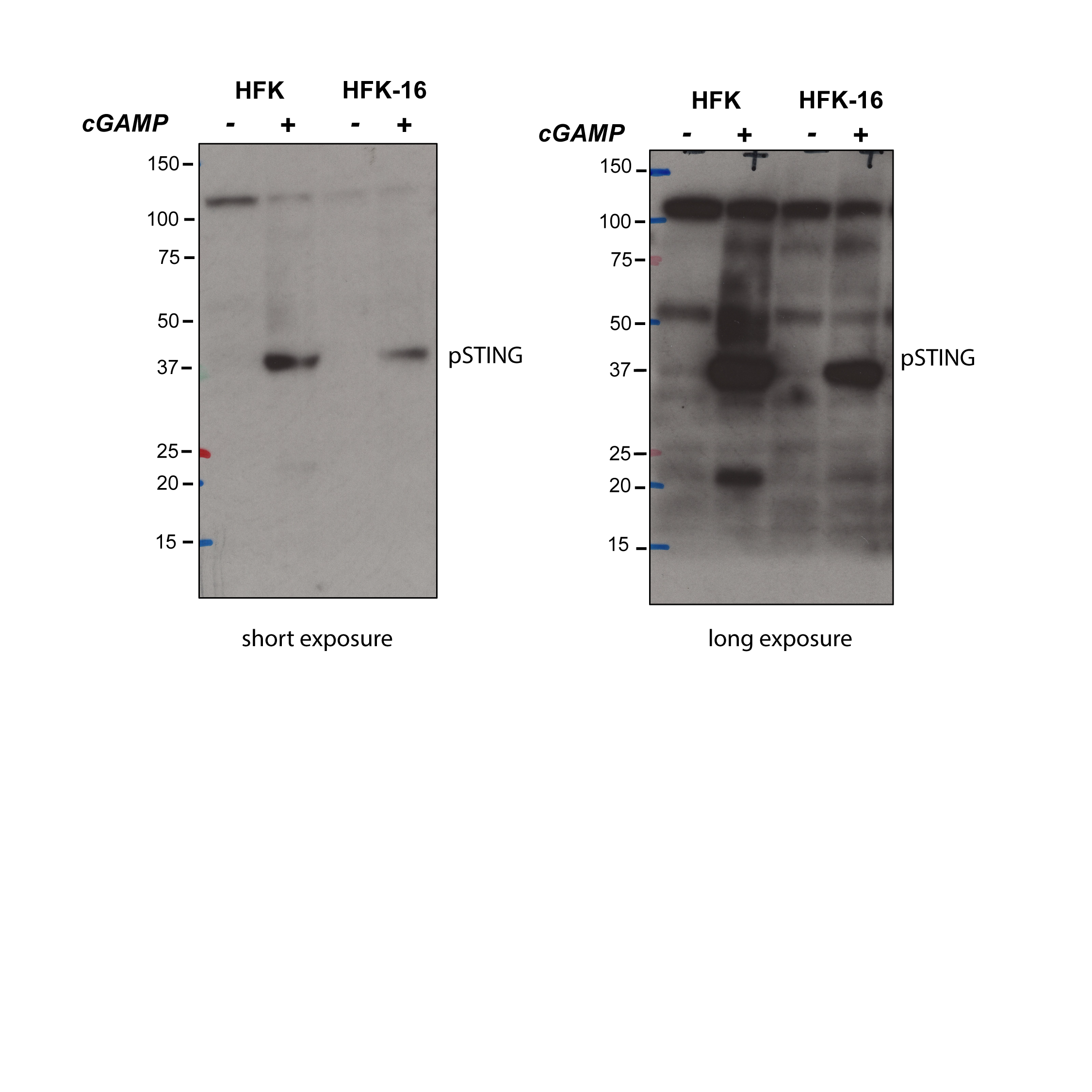

Supplement: S6 Fig — The entire blots for pSTING bands shown in Fig 2A for short and long exposures are presented. (TIF) [file ppat.1010725.s006.tif]

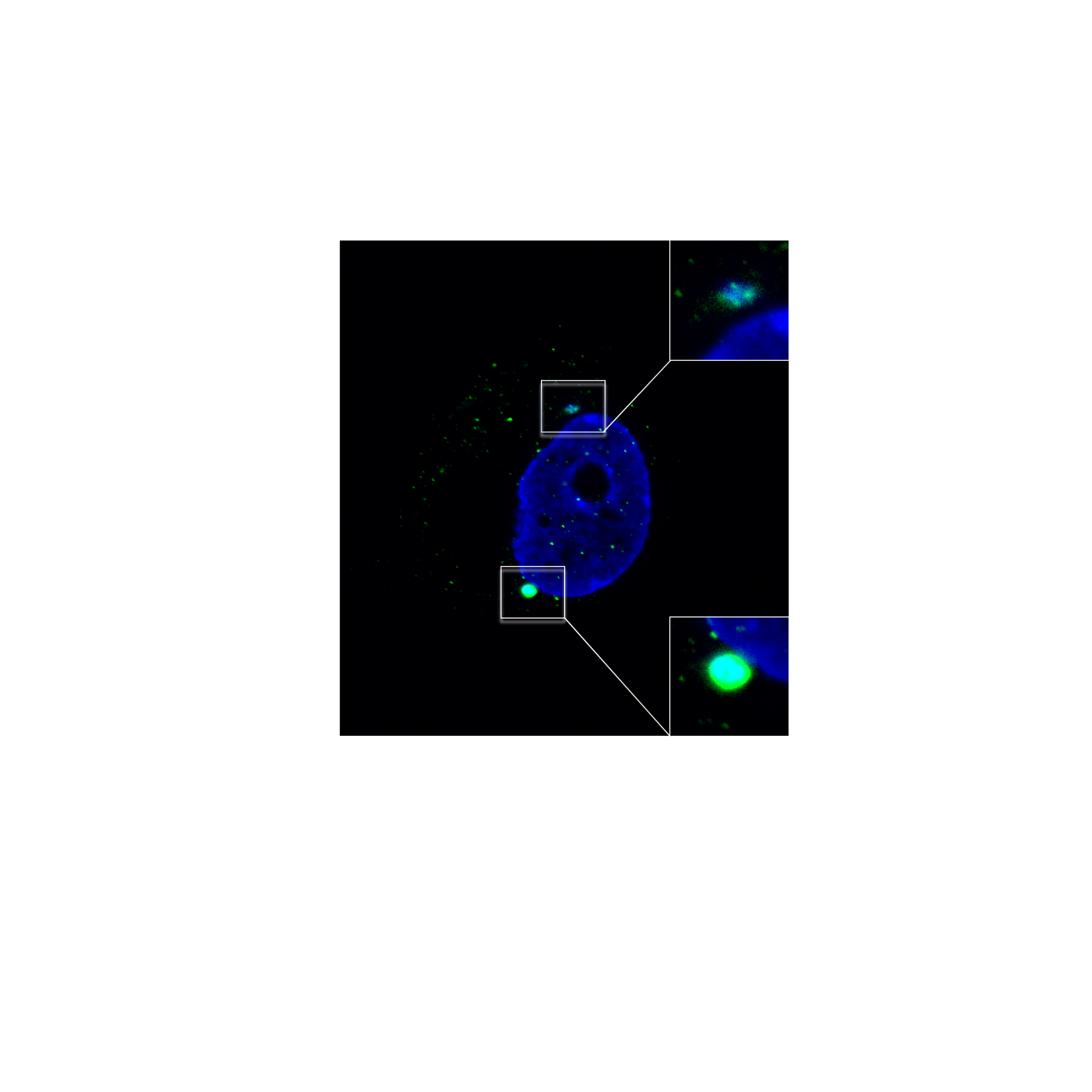

Supplement: S7 Fig — Higher intensity exposure of CIN612 ctrl panel in Fig 6 post etoposide treatment showing nuclear and cytosolic foci along with staining in a second micronucleus. A close-up image of the two micronuclei in this cell is also included. (TIF) [file ppat.1010725.s007.tif]
